# Mathematical Modeling of the Function of Warburg Effect in Tumor Microenvironment

**DOI:** 10.1038/s41598-018-27303-6

**Published:** 2018-06-11

**Authors:** Milad Shamsi, Mohsen Saghafian, Morteza Dejam, Amir Sanati-Nezhad

**Affiliations:** 1 Center for BioEngineering Research and Education, University of Calgary, Calgary, Alberta T2N 1N4 Canada; 2 Department of Mechanical Engineering, Isfahan University of Technology, Isfahan, 8415683111 Iran; 3 Department of Petroleum Engineering, College of Engineering and Applied Science, University of Wyoming, 1000 E. University Avenue, Laramie, Wyoming 82071–2000 USA; 4 BioMEMS and Bioinspired Microfluidic Laboratory, Department of Mechanical and Manufacturing Engineering, University of Calgary, Calgary, Alberta T2N 2N1 Canada

## Abstract

Tumor cells are known for their increased glucose uptake rates even in the presence of abundant oxygen. This altered metabolic shift towards aerobic glycolysis is known as the Warburg effect. Despite an enormous number of studies conducted on the causes and consequences of this phenomenon, little is known about how the Warburg effect affects tumor growth and progression. We developed a multi-scale computational model to explore the detailed effects of glucose metabolism of cancer cells on tumorigenesis behavior in a tumor microenvironment. Despite glycolytic tumors, the growth of non-glycolytic tumor is dependent on a congruous morphology without markedly interfering with glucose and acid concentrations of the tumor microenvironment. Upregulated glucose metabolism helped to retain oxygen levels above the hypoxic limit during early tumor growth, and thus obviated the need for neo-vasculature recruitment. Importantly, simulating growth of tumors within a range of glucose uptake rates showed that there exists a spectrum of glucose uptake rates within which the tumor is most aggressive, i.e. it can exert maximal acidic stress on its microenvironment and most efficiently compete for glucose supplies. Moreover, within the same spectrum, the tumor could grow to invasive morphologies while its size did not markedly shrink.

## Introduction

Upregulation of glucose uptake rate and its fermentation in anaerobic pathways, even under normoxic conditions, is a prevalent observation in cancer cells^1^. This phenomenon, known as Warburg effect, has brought about an enormous amount of research aimed at explaining how the altered glucose metabolism benefits cancer cells. A number of possible advantages of Warburg effect offered to cancer cells include enhanced biosynthetic activity, accelerated adenosine triphosphate (ATP) production, altered cell signaling, and a reduced risk of reactive oxygen species (ROS) mediated damage to malignant cells^2–4^. Another explanation for the benefit of Warburg effect in the tumor microenvironment is related to the condition where cancer cells utilize aerobic glycolysis to increase their acid production and invade the adjacent healthy tissue^5^. Experimental evidence also shows that peritumoral regions with higher acidity exhibit usually stronger invasive potential than regions with normal extracellular pH^6^. Moreover, it has been proposed that the microenvironmental acidity helps tumors to evade immune surveillance^7^. Furthermore, an increase in glucose uptake rate by tumors has been claimed to blunt immune performance by depleting microenvironmental glucose^8,9^.

Mathematical modeling has been extensively utilized to explain the Warburg effect. Many of these mathematical models (reviewed elsewhere)^10^ have explained the advantages of the Warburg effect in terms of accelerated ATP production and enhanced biosynthetic activity. The reason for the shift toward fermentation, in spite of the fact that the respiration pathway is more efficient in terms of ATP yield per mole glucose, has also been tackled mathematically. Using the flux balance analysis (FBA) method, Vazquez *et al*.^11^ attributed metabolic reprogramming towards fermentation in highly glycolytic cells to a limited intracellular space available to mitochondrial enzymes. There have also been efforts to explain the logic of Warburg effect within the framework of evolutionary game theory (EGT)^12–15^. The EGT models the co-evolution of cells in the tumor microenvironment as a game with cells as players. The outcome of this game is determined by the strategies (e.g. glycolysis or oxidative phosphorylation) that different cell phenotypes adopt^15^. Kareva^13^ and Archetti^14^ used EGT to explain the rise of a population of glycolytic cells in the tumor as a trade-off between the cost (low ATP yield per glucose) and the benefit (toxification of normal cells) harbored by aerobic glycolysis.

Gatenby and Gawlinski^16^ formulated the hypothesis of acid-mediated tumor invasion with a reaction-diffusion mathematical model. Their model recapitulated the dynamics of tumor-host interface and gradients of microenvironmental pH, and predicted a correlation between tumor-host morphology and tumor growth rate. Patel *et al*.^17^ utilized a hybrid continuum-discrete model of tumor microenvironment to assess the impact of normal tissue vascularity on early tumor growth and invasion. It was shown that there exists a definite range of tissue vascularity for each glucose uptake rate of tumors with highest growth advantages to tumor cells. Moreover, the impact of normal tissue vascularity on the growth of large tumors was assessed by utilizing a fully continuum tumor model^18^. The results proved the essence of vascularization in an avascular tumor to avoid auto-toxicity with therapeutic implications for targeting the tumor vasculature. A subsequent study integrated *in vivo* experiments in the dorsal skinfold chamber with an *in silico* model of acid-mediated invasion to predict peritumoral tissue remodeling^19^. In line with the findings of Gatenby and colleagues^19^, EGT simulations in the context of gliomas have shown that the presence of glycolytic cancer cells in the tumor microenvironment can facilitate the emergence of invasive phenotypes^12^. Coupling the cellular automaton model with transport equations of chemicals, Smallbone *et al*.^20^ proposed that the emergence of glycolytic and acid resistant cancer cells enables breast cancer cells to compete with normal epithelial cells and invade the basal membrane. Experimental data from tumor spheroids and clinical specimens also highlighted the role of acid resistant and glycolytic phenotypes of cancer cells in invasiveness of breast cancer^21^.

The metabolism-mediated immune escape as a result of glucose competition between malignant cells and the lymphocytes was modeled as a set of ordinary differential equations^22^. The glucose competition was shown to lead to oscillatory tumor growth modes which could be proceeded by immune escape. Direct interference with microenvironmental pH to perturb the tumor acidic milieu has also been the subject of a number of studies^23–26^. Silva *et al*.^24^ developed a computational model to predict the efficacy of the so called “pH buffer therapy”. Simulation results suggested that oral administration of sodium bicarbonate (NaHCO3) could alleviate microenvironmental acidity. Later, Martin *et al*.^23^ combined data from *in vivo* animal models with simulation results to predict the efficacy and safety of pH buffer therapy for humans. Recent studies within the EGT framework revealed that intratumoral metabolic heterogeneity can give rise to resistance to pH targeting therapies, and that normalizing microenvironmental oxygen levels (e.g. via vascular normalization therapy) could improve pH buffer therapies in metabolically heterogeneous tumors^25,26^. Moreover, mathematical modeling also suggests the administration of vascular normalization therapy prior to pH targeting as the optimal sequence to treat heterogeneous tumors^26^.

Despite extensive previous modeling, the detailed effects of glucose metabolism of cancer cells on tumor growth is unknown. Through systematic analysis we explore how exactly the glucose metabolism of cancer cells shapes tumor behavior. A computational model is used to screen morphology, vascularity, and cellularity of tumors with different glucose uptake rates over time. We show that upregulated glucose metabolism can help a tumor propagate in a poorly vascularized tissue without to rely on neo-vasculature recruitment. Hence, the success of anti-angiogenic therapies aimed at starving tumors depends on the glucose uptake rate of cancer cells. The model is also used to depict the trade-off between acid production and competition for limited supplies of glucose in tumors with different glucose uptake rates. Furthermore, we identify, for the first time, a spectrum of glucose uptake rates within which glycolytic tumors emerge to be most hostile. Within this spectrum, tumors can effectively compete over glucose resources, maximally acidify their microenvironment, and grow to invasive morphologies while they pay only little price in terms of cellularity and size reduction. Overall, our results imply that the glucose uptake rate of the tumor could serve as a prognostic factor for patient survival and a biomarker for the outcome of anti-angiogenic therapeutics.

## Results

We developed a four-compartment model to simulate the growth of tumors with variable glucose uptake rates. The model incorporates the dynamics of cancer cells, sprouting angiogenesis, hemodynamics, and the transport of diffusible chemicals in a tumor microenvironment. Each model compartment is explained in details in the Methods section. In the “cancer cell dynamics” compartment of the model, the glucose uptake rate of cancer cells is computed by Equation (4). In Equation (4), the parameter *p*_*G*_ is used to model the Warburg effect, and the glucose uptake rate of each tumor is represented by a certain value of *p*_*G*_. While *p*_*G*_ = 1 denotes a normal glucose uptake rate, upregulated glucose metabolism is modeled by assigning a value greater than unity to the parameter *p*_*G*_. The value of *p*_*G*_ varied in the range of 1 ≤ *p*_*G*_ ≤ 50 as reported in the recent work of Robertson-Tessi *et al*.^27^ Examining Equation (4), it was realized that the setting of *p*_*G*_ = 50 increases glucose uptake rate by a factor of almost 750. It has been experimentally proven that tumors can upregulate glucose uptake rates up to three orders of magnitude^27,28^. To probe glucose uptake rates more exhaustively, *p*_*G*_ was considered to vary in the range 1 ≤ *p*_*G*_ ≤ 100. Next, we present the simulation results for non-glycolytic (*p*_*G*_ = 1) and glycolytic (1 < *p*_*G*_ ≤ 100) tumors.

### Non-glycolytic tumor

#### The non-glycolytic tumor induces angiogenesis and grows to non-invasive morphologies

**Figure 1:**
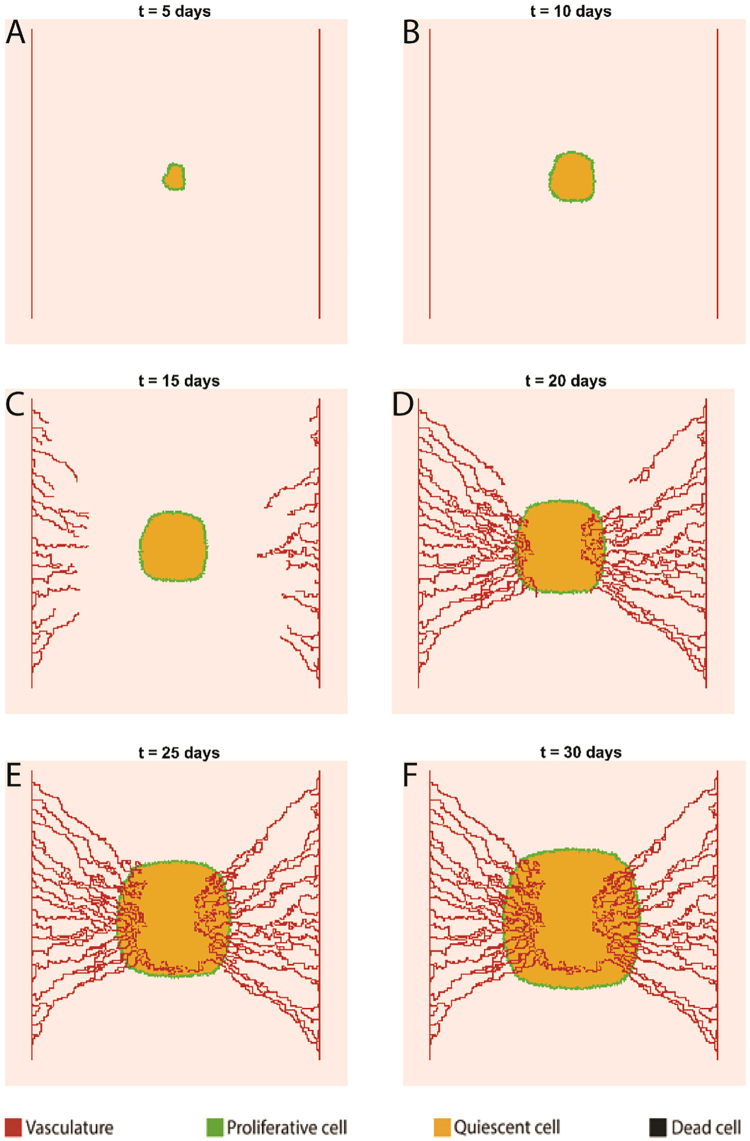
Non-glycolytic tumor growth and angiogenesis simulated over a 30-day period. **(A,B)** Snapshots of tumor and adjacent parent vessels in 5 and 10 days, respectively, prior to the induction of angiogenesis. **(C)** Tumor and vasculature status at day 15 shortly after the initiation of angiogenesis. **(D–F)** The evolution of tumor and establishment of perfused vascular loops from day 20 onwards. The tumor does not exhibit any fingering in the absence of Warburg effect.

Starting with three cancer cells at *t* = 0, the tumor mass at day 5 consists of a core of quiescent cells encompassed with a rim of proliferative cells (Fig. 1A). Cancer cells with completed cycles at the central area of the tumor become quiescent due to the lack of space. The tumor retains its overall morphology (i.e. the quiescent core and proliferative rim) by day 10 (Fig. 1B) while it grows from a diameter of 40 µm (at *t* = 0) to a diameter of almost 800 µm. When the tumor size reaches 1 mm in diameter, the oxygen supply from the two parent vessels can no longer nourish the tumor mass. Hence, hypoxic regions with P_O2_ < 10 mmHg are initiated within the tumor and hypoxic tumor cells start secreting tumor angiogenic factors such as vascular endothelial growth factor (VEGF). The secreted VEGF diffuses into the interstitium and initiates angiogenic sprouting on the adjacent parent vessels. Thus, capillary sprouts are present in the tumor microenvironment at day 15 (Fig. 1C). Nascent sprouts further elongate, branch, and anastomose as they migrate in the direction of VEGF gradients (via chemotaxis) while interacting with the ECM macromolecule fibronectin (via haptotaxis). Microvascular loops that convey blood flow are visible in Fig. 1D,E. The loops deliver blood borne nutrients like glucose and oxygen to tumor cells while they carry away metabolic waste (H^+^), and therefore prevent tumor acidosis and starvation. Therefore, tumor growth is fully supported by the nascent microvascular network as time further elapses toward day 30 (Fig. 1E). At day 30, the tumor reaches a diameter of almost 2 mm and consists of a well vascularized proliferative rim and a quiescent core. It is noteworthy that no region with dead cells are observed at the end of the 30-day period in a non-glycolytic tumor. Hence, it is concluded that neither tumor ATP reduction nor microenvironmental acidification could induce cell death. Another aspect of non-glycolytic tumor growth is the preservation of the circular shape of the tumor throughout the growth period, which is discussed in more details in the non-glycolytic tumor model.

**Figure 2:**
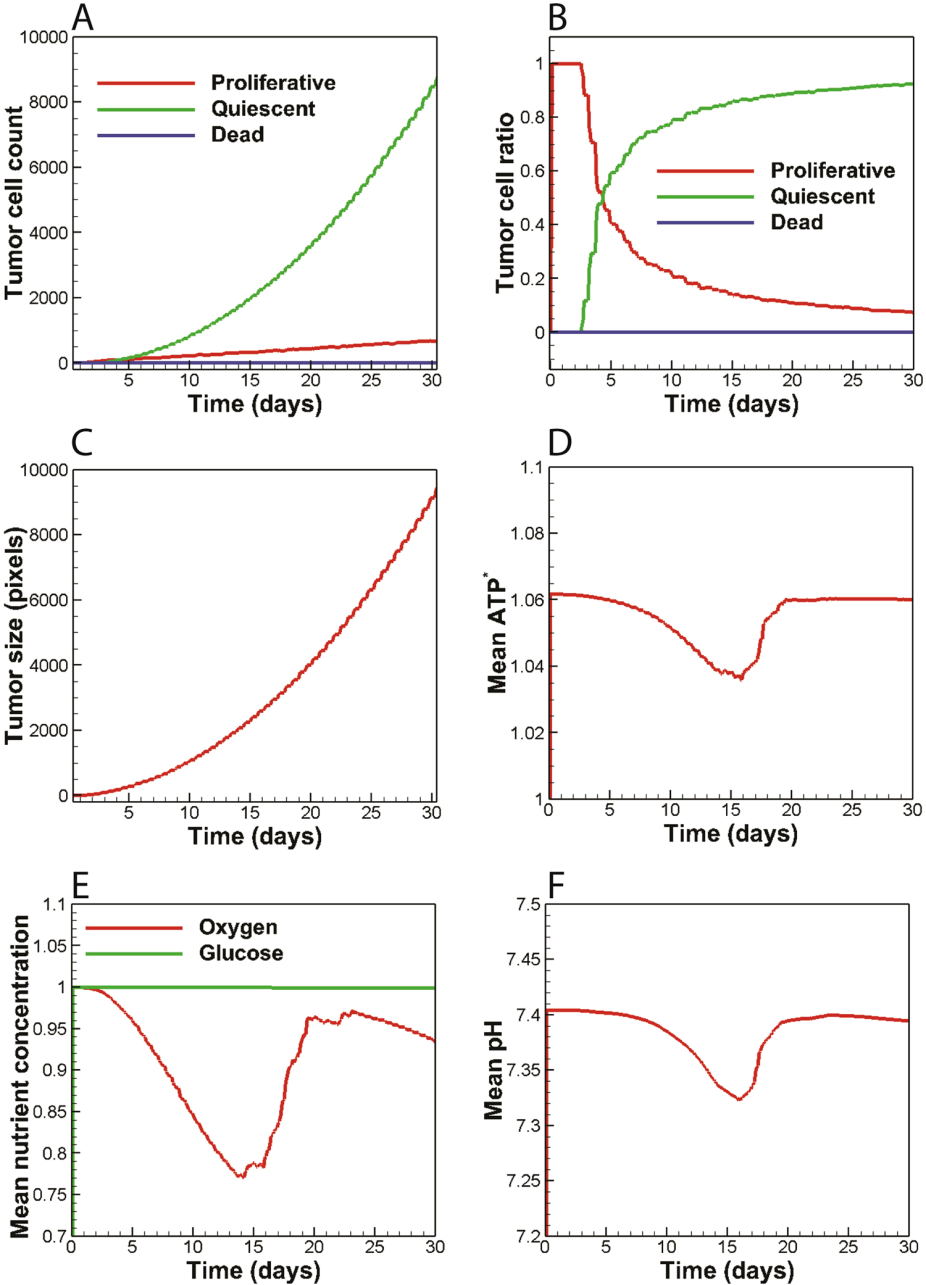
The variables of non-glycolytic tumor plotted versus time. (**A**) The number of proliferative and quiescent cancer cells grows in linear and non-linear modes, respectively; while the number of dead cells remains unchanged throughout. (**B,C**) The tumor grows exponentially and the population ratios of cancer cells grow asymptotically to a fixed value before day 30. (**D**) The tumor ATP yield reduces by 2.4% due to nutrient depletion and is restored to normal levels after angiogenic switch. (**E)** Mean oxygen concentration is lowered below the hypoxic and later restored via recruitment of neo-vasculature while glucose content remains almost intact. **(F)** Microenvironmental pH drops up to 0.1 units and is later restored to normal values due to the existence of neo-vasculature.

The temporal evolution of tumor cell count and tumor cell population ratio is presented in Fig. 2A,B, respectively. The proliferative cell count tends to increase linearly while exhibiting a relatively small growth rate. The number of quiescent cancer cells, on the other hand, increases nonlinearly with a much faster rate than proliferative cells. Therefore, the ultimate number of quiescent cells at the end of the simulation time is remarkably higher than the proliferative cells. It is also noted that the number of dead cells remains unchanged. All cancer cells at t = 0 are observed in the proliferative state (Fig. 2B). After three days, the lack of space is observed for a number of proliferative cells located in the tumor core, resulted in their turn to a quiescent state. The growth of tumor further affects other cells due to the lack of space and thus a greater percentage of cells become quiescent. As a result, the proliferative cell ratio experiences a steep decline while the quiescent cell ratio increases rapidly. This behavior leads to an equal number of cells in proliferative and quiescent states at day 5. From day 5 onwards, the overall trends of the proliferative and quiescent curves are preserved, although the rate of changes in cell count reduces gradually. Finally, at about day 30, the ratios of cancer cell populations become saturated, where the quiescent cells constitute 93% of the tumor cell population while the proliferative cells make up only 7% of the tumor mass. The overall behavior of cancer cell diagrams observed in Fig. 2A,B was also recapitulated by another numerical study^29^. Also the tumor size grows exponentially as observed in Fig. 2C. Moreover, the mean ATP production of cancer cells declines by only 2.4% as a result of nutrient depletion (Fig. 2D). However, cancer cells retrieve their normal ATP production when they are nourished with nutrients supplied by neo-vasculature.

#### The non-glycolytic tumor has little impact on microenvironmental glucose and pH levels while it depletes oxygen

Temporal evolution of mean metabolite concentrations in the tumor microenvironment is depicted in Fig. 2E,F. Oxygen and glucose concentrations were non-dimensionalized with the reference values *C*_*O2,0*_ = *5.2* × 10^*−6*^ *mol/L* and *C*_*G,0*_ = *5.0* × 10^*−3*^ *mol/L*, respectively. Moreover, the acid concentration was reported in the well-known pH scale. A non-glycolytic tumor consumes oxygen as its nutrient and does not cause significant changes in the microenvironmental glucose content (Fig. 2E). Tumor cells consume oxygen and duplicate until the supply of oxygen provided by parent vessels is no longer able to maintain the oxygen tension above the hypoxic limit. As a result, hypoxic cells seek to recruit neo-vasculature from adjacent parent vessels via sprouting angiogenesis. From day 14 onwards, oxygen delivery by nascent vasculature tends to restore the microenvironmental oxygen levels, and thus the mean oxygen concentration continuously increases until day 18. Afterwards, oxygen consumption by an enlarged tumor dominates the oxygen delivery by tumor vasculature, and therefore oxygen levels start reducing once again. Since the tumor vasculature acts as a sink for hydrogen ions, the trend observed in Fig. 2F could also be explained as to that observed in Fig. 2E. Starting with an initial normal pH value of 7.4 in tumor microenvironment, pH levels exhibit a 0.1 unit drop by the end of day 10. Next, the angiogenic switch brings about perfused vessels that carry away metabolic waste, albeit with a 3-day delay. pH level then increases after the emergence of interconnected microvessels and stabilizes completely by day 18. The tumor vasculature then acts as a sink for metabolic waste and preserves the microenvironmental pH levels.

### Glycolytic Tumor

#### Warburg effect induces invasive tumor morphology while it reduces tumor size

**Figure 3:**
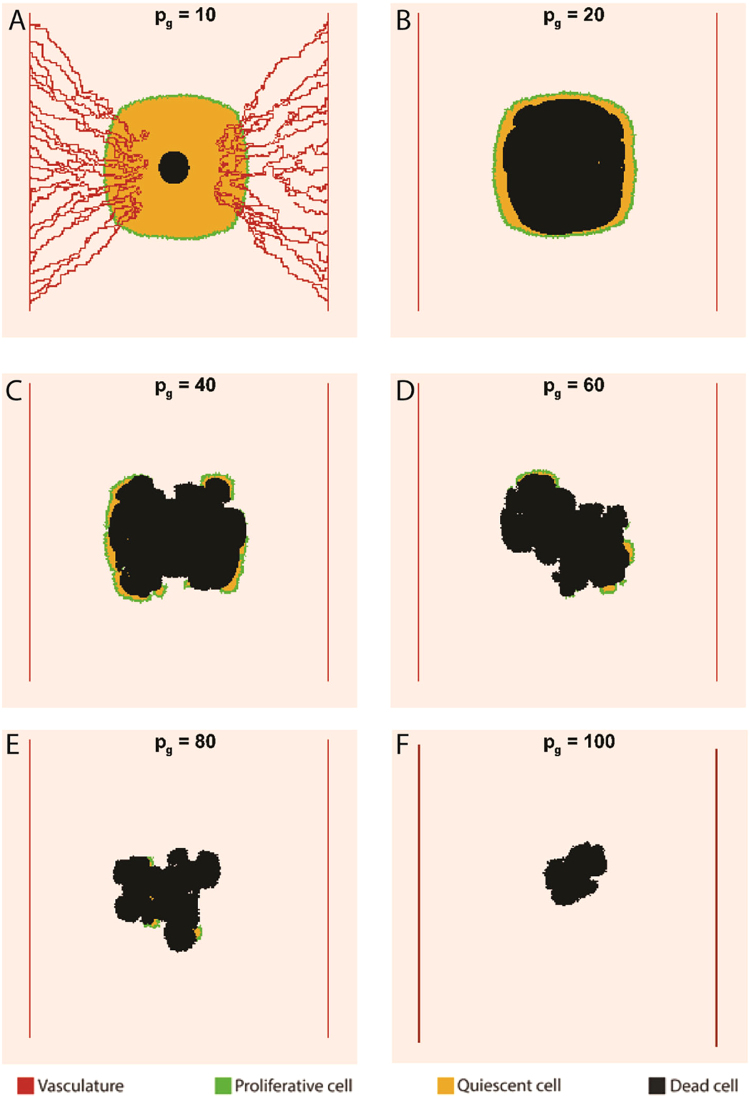
The effect of Warburg effect on tumor morphology at day 30. **(A)** The tumor with *p*_*G*_ = 10 instigates angiogenesis. **(B)** The size of dead core increases as *p*_*G*_ reaches 20. **(C,D)** The tumors start invasive morphologies as *p*_*G*_ exceeds 20. **(E,F)** The excessive reliance on aerobic glycolysis leads to a size reduction due to acidosis.

**Figure 4:**
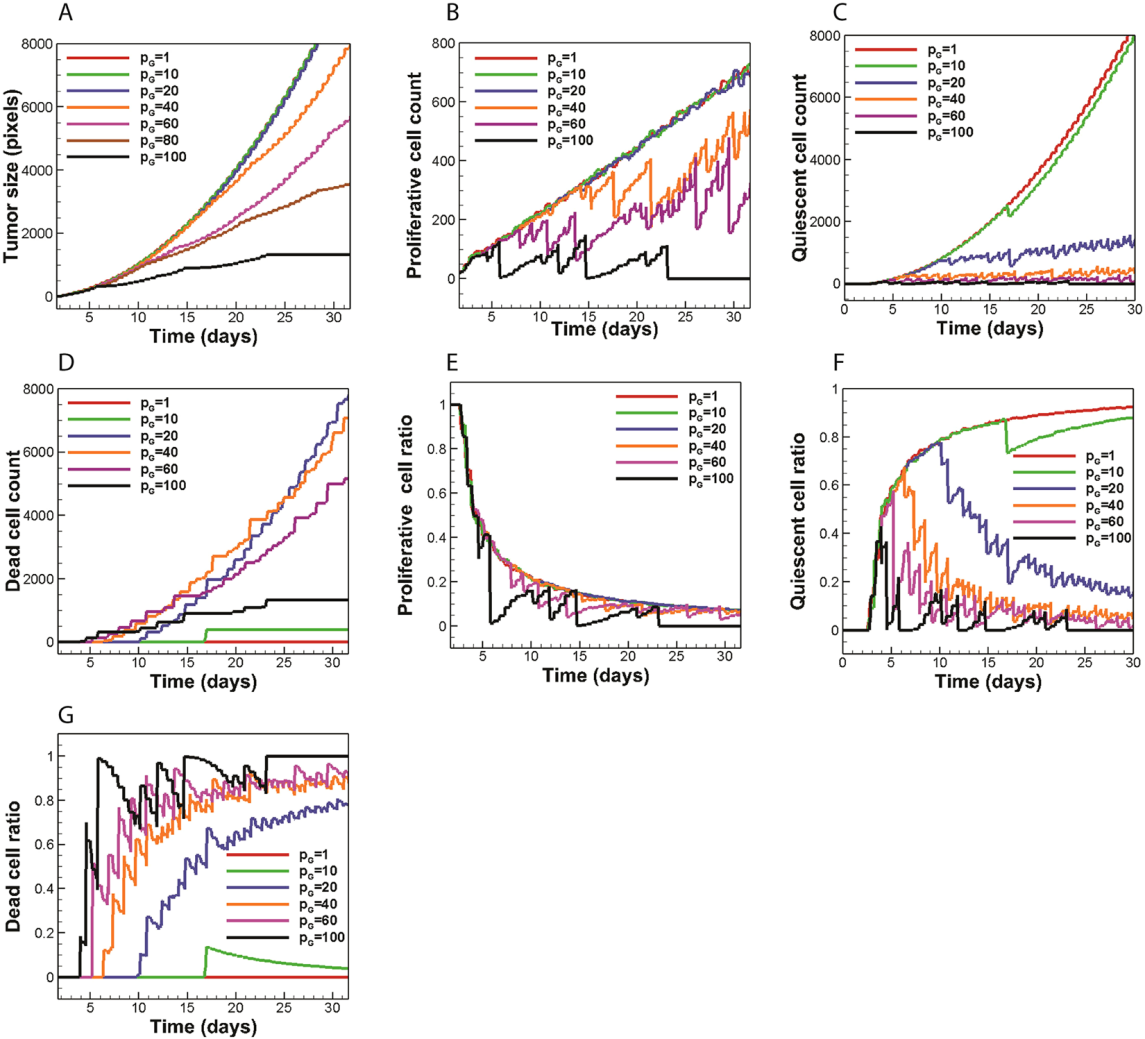
The role of Warburg effect on glycolytic tumor variables. **(A)** The Warburg effect tends to reduce tumor size. **(B**) Increasing *p*_*G*_ to values below 20 has little influence on proliferative cell count. Proliferative cell count tends to reduce for ${p}_{G}\ge 40$. **(C)** The number of quiescent cells decreases due to the consumption of oxygen by cancer cells. **(D)** Dead cell count exhibits an initial increase as *p*_*G*_ increases to a certain value and drops afterwards. **(E–G)** Proliferative and quiescent cells constitute a smaller fraction of the tumor as the glucose uptake is upregulated and dead cells tend to replace the proliferative and quiescent cells in the body of tumor. Fluctuations in population curves are more intense at higher values of *p*_*G*_.

Figure 3 shows the impact of Warburg effect on tumor morphology at day 30. In contrast to the non-glycolytic tumor, glycolytic tumors include dead regions. Also, the round shapes of tumors shift toward invasive geometries as the reliance on aerobic glycolysis increases. While tumors with *p*_*G*_ = 10 and 20 exhibit relatively round shapes, tumors with *p*_*G*_ ≥ 40 begin to exhibit invasive morphologies. Meanwhile, the size of tumor shrinks as the Warburg effect becomes stronger. The impact of Warburg effect on tumor size is presented in Fig. 4A. Initially, all tumors are observed to have the same size but the curves with different values of *p*_*G*_ start diverging from each other over time. The higher the value of *p*_*G*_, the sooner the curve diverges from other curves. For smaller values of *p*_*G*_, the tumor size is less sensitive to changes in glucose uptake rates. For example, a 20-fold increase in *p*_*G*_ from unity to 20 has a little effect on the tumor size but as *p*_*G*_ increases above 20, the effect of glucose uptake rate on tumor size becomes noticeable. Thus, a 5-fold increase in *p*_*G*_ from 20 to 100 causes a 59% reduction in tumor size at day 15. The significant reduction in the size of the highly glycolytic tumor (*p*_*G*_ = 100) stems from tumor acidosis and ultimately leads to a thorough growth cessation at day 23.

Further quantitative data on cancer cell population dynamics is shown in Fig. 4B–G. The results show no impact on proliferative cell count when *p*_*G*_ increases ten folds from unity while it brings about a local shift in the quiescent cell count at day 17 due to the occurrence of acid-induced death in the interior of the tumor (Fig. 4B,C). Moreover, increasing the value of *p*_*G*_ to 20 slightly affects proliferative cell count while it markedly alters the number of quiescent and dead cells. According to Fig. 3B, for *p*_*G*_ = 20, the outer proliferaive rim is preserved while quiescent cells located in the core of the tumor are subject to acid-induced death. Thus, an exclusive change in quiescent cell count takes place. As *p*_*G*_ reaches 40, the number of proliferative cells starts fluctuating strongly with time. This fluctuating behavior is the result of acid accumulation in the tumor microenvironment (which frequently kills a number of cancer cells) and is also observed in the proliferative cell count of tumors with *p*_*G*_ ≥ 40. The number of dead cells in the tumor with *p*_*G*_ = 10 changes from zero to 400 at day 17, and remains constant thereupon (Fig. 4D). The fixed dead cell count in the tumor with *p*_*G*_ = 10 is due to the efficient removal of acids by tumor vasculature which prevents further cell death by acidosis. The tumor with *p*_*G*_ = 20 has the most number of dead cells at day 30 which seems to be contradictory as the tumors with *p*_*G*_ > 20 produce more acid and are more inclined to acid-induced cell death. However, a closer look at the curves of dead cell count shows that tumors with *p*_*G*_ > 20 possess non-zero values earlier than the tumor with *p*_*G*_ = 20. Hence, there are less proliferative tumor cells in the tumor body in subsequent time points. As a result, tumors with *p*_*G*_ > 20 have smaller sizes compared to the tumor with *p*_*G*_ = 20, meaning that there exist less cancer cells prone to acid-induced cell death in tumors with *p*_*G*_ > 20. Consequently, the tumor with *p*_*G*_ = 20 shows a larger body of dead cells by the end of day 30.

The overall behavior of proliferative cell population ratio is preserved as the value of *p*_*G*_ increases (Fig. 4E). Nonetheless, cellular population ratios tend to fluctuate as the glucose uptake rate increases. The greater the value of *p*_*G*_, the stronger the fluctuation in cellular population ratio. At the limit of *p*_*G*_ = 100, the fluctuations become so severe that the proliferative cell population ratio falls to zero before day 30 (i.e. thorough tumor acidosis occurs). It is concluded that extremely high values of aerobic glycolysis could destabilize the population of proliferative cells. In contrast to the proliferative cell population ratio, the overall behavior of quiescent cell population ratio is not retained as the tumor shifts from a non-glycolytic regime to a glycolytic one (Fig. 4F). Following an early rise in the non-glycolytic trend, the quiescent cell population ratio decreases toward a glycolytic trend. The higher the value of *p*_*G*_ the sooner the diversion occurs. The impact of glucose metabolism on dead cell population ratio is also shown in Fig. 4G. Even though the number of dead cells decreases in response to the increase in glucose uptake rate (Fig. 4D), dead cells constitute a greater ratio of the tumor body for *p*_*G*_ > 20.

#### Acid production regulates tumor glucose competition and neo-vascularization

**Figure 5:**
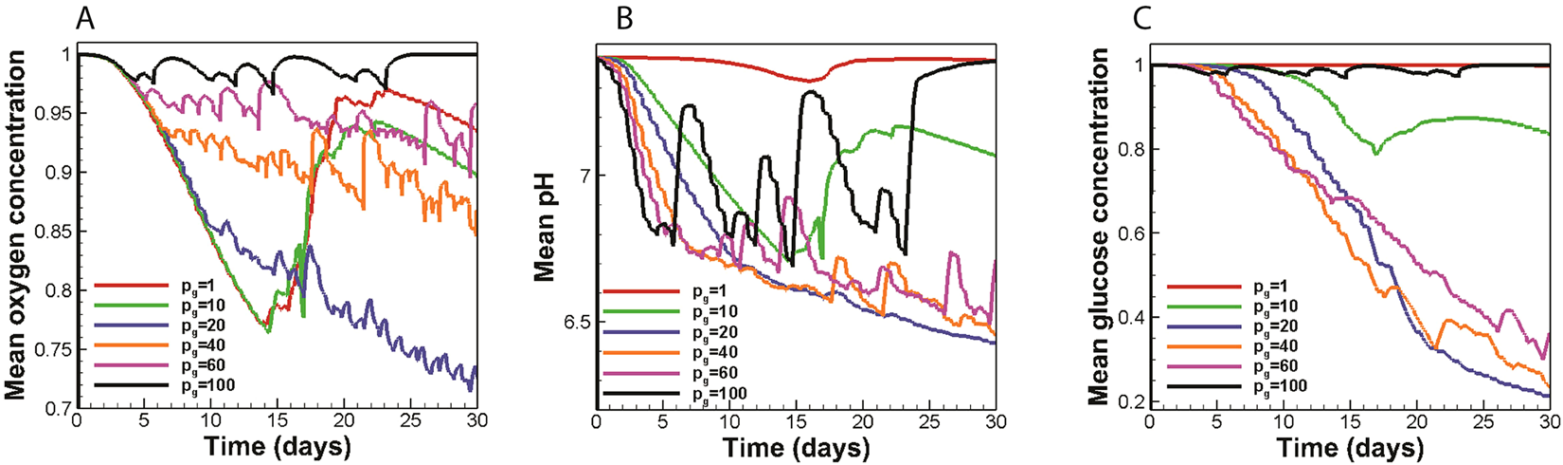
The impact of Warburg effect on nutrient availability and microenvironmental acidity. **(A)** For tumors with ${p}_{G}\ge 20$, the more glycolytic a tumor is, the better it retains oxygen levels above the hypoxic limit. **(B)** The tumor with *p*_*G*_ = 20 exerts maximal acidic stress on its microenvironment. **(C)** The tumors with $20\le {p}_{G}\le 40$ most effectively compete for glucose resources.

A remarkable trend observed in Fig. 3 is related to tumor vascularity. Only the tumor with *p*_*G*_ = 10 relies on angiogenesis to maintain its growth. The impact of Warburg effect on the temporal evolution of mean oxygen concentration is shown in Fig. 5A. The oxygen curve of the glycolytic tumor with *p*_*G*_ = 10 shares the same trends as the non-glycolytic tumor. Since the tumor with *p*_*G*_ = 10 induces angiogenesis, an initial oxygen reduction is accompanied by tumor oxygenation. However, Fig. 5A also shows that higher rates of glucose uptake lead to less oxygen depletion by the tumor, which justifies the absence of angiogenesis in tumors with *p*_*G*_ ≥ 20. In other words, glycolytic tumors tend to retain normal oxygen levels in the tumor microenvironment. According to Fig. 5A,B, the maintanance of oxygen levels is associated with frequent acidosis. Cellular population fluctuatuions observed for tumors with *p*_*G*_ ≥ 20 (Fig. 4B–G) also indicate that the population of oxygen consuming cells is is subject to frequent acid induced death. Therefore, it is inferred that glycolytic tumors with *p*_*G*_ ≥ 20 can propagate economically under limited nutrient availability via frequent self-toxification. This management of resources through self-toxification has strong therapeutic implications as it suggests that anti-angiogenic therapeutics aimed at starving the tumor may not benefit patients with highly glycolytic tumors (*p*_*G*_ ≥ 20). It is concluded that supplementing current anti-angiogenic therapeutics with treatment modalities that control tumor acidity (via either targeting the Warburg effect or augmenting the buffering capacity of the tumor microenvironment) could lead to improved efficacy.

Depletion of limited microenvironmental glucose resources by the tumor serves as a useful strategy for cancer cells to hinder the performance of immune cells^8,9^. The impact of Warburg effect on mean glucose content of the tumor microenvironment is shown in Fig. 5C. Strikingly, it is observed that tumors with higher glucose uptake rates are not necessarily better glucose competitors. In fact, tumors with 20 ≤ *p*_*G*_ ≤ 40 can most effectively deplete microenvironmental glucose content. The trend observed in the glucose concentration curve seems to be counter intuitive at first glance but it could be explained as the trade-off between two opposing factors. Firstly, increasing the *p*_*G*_ value leads to more glucose consumption and a tendency to lower the glucose content. Secondly, an increase in *p*_*G*_ is associated with more acid production and elevated acid-induced cell death (which means that there is less tumor cells to compete for glucose resources). For *p*_*G*_ ≤ 40, the former effect is dominant, and the net glucose consumption rate is enhanced as the glucose uptake rate increases. For *p*_*G*_ > 40, on the other hand, acidic cell death becomes dominant and the glucose content is preserved.

#### Tumors with intermediate values of p_G_ are the most aggressive tumors

According to Fig. 5B, the tumor with *p*_*G*_ = 20 is found to reduce the pH of the tumor microenvironment by 1.1 units in a steady manner. Moreover, even though there exist some pH fluctuations during the last 13 days of tumor growth, the tumor with *p*_*G*_ = 40 can also exert remarkable acidic stress on its microenvironment and reduce the mean pH level up to 0.94 units. Moreover, tumors with 20 ≤ *p*_*G*_ ≤ 40 can most effectively deplete microenvironmental glucose content (Fig. 5C). The tumors also take on invasive morphologies for 20 < *p*_*G*_ ≤ 40 while they pay little price in terms of size reduction. For instance, the size of tumor with *p*_*G*_ = 40 (at day 30) decreases by only 19% compared to the size of the non-glycolytic tumor (Fig. 4A). Meanwhile, compared to the non-glycolytic tumor, the number of proliferative cancer cells (necessary for tumor propagation) reduces by almost 25% and their population ratio reduces by only 5.4% at day 30 (Fig. 4B,E). Glucose depletion and microenvironmental acidity lead to an immune evasion. Invasive tumor morphologies are also associated with poor therapeutic outcome. Taken together, it is inferred that there exists an intermediate range of 20 < *p*_*G*_ < 40 for the tumors considered in this study that makes them the most troublesome from a therapeutic point of view. These tumors can potently compete over glucose resources and acidify their microenvironment, grow to invasive morphologies, and propogate without the need for angiogenesis while they pay only little price in terms of cellularity and size.

## Discussion

Extensive experimental data provides compelling evidence that the Warburg effect can be held responsible for acid-mediated tumor invasion and immune suppression^6,8,30^. Moreover, tumor aerobic glycolysis adversely affects the outcome of therapeutic interventions such as chemotherapy and immunotherapy^31,32^. The Warburg effect can be associated with dismal patient prognosis^33,34^. Hence, it is imperative to grasp a better understanding of this phenomenon. The existing *in vivo* tumor models are not compatible with systemic analysis of phenomena in tumor microenvironment. On the other hand, current *in vitro* assays, while highly controllable, are costly and unable to fully model the complexity of the tumor microenvironment. On the contrary, computational models can act as a potent, cost-effective tool to dissect tumor microenvironment as a complex system^35,36^. We developed a computational model of tumor microenvironment that could follow the growth of tumors with different glucose uptake rates in both vascular and avascular phases. The results were used to show how the so-called Warburg effect modulates tumor microenvironment and affects tumor growth. The model parameter *p*_*G*_ was used to characterize the glucose uptake rate of tumors.

One important issue illustrated in this work is the ability of glycolytic tumors to progress in poorly vascularized tissues without the need to rely on neo-vasculature. This finding can have therapeutic implications in terms of the relation between the glucose metabolism of a tumor and its resistance to anti-angiogenic therapies. Recent experimental studies also corroborate the correlation between the glucose metabolism of tumors and outcome of anti-angiogenic therapies^37,38^. According to these studies, despite the shortfall in glucose resources, highly glycolytic tumor xenografts develop extensive regions of dead cells and become ultimately resistant to VEGF blockade. On the other hand, poorly glycolytic xenografts remain mostly viable in the short term while they undergo remarkable size reduction in the long term. A significant prediction of our *in silico* tumor model is the existence of a certain range of glucose uptake rates (characterized by 20 < *p*_*G*_ ≤ 40) of tumors within which the tumor exhibits remarkable aggression by maximally acidifying its milieu, competing effectively over glucose resources, and taking on invasive morphologies. Based on Equation (4), the *p*_*G*_ range found in this study corresponds to a 300–600 folds increase in glucose uptake rate. Given the capabilities of current imaging facilities, this prediction can be further examined by determining the tumor glucose uptake rates of several patients (via ^18^fluorodeoxyglucose-positron emission tomography (FDG-PET)) and comparing the morphology, size and cellularity of tumors over time^39,40^.

In this work, we modeled early tumor growth in a poorly vascularized tissue. The model can be extended to account for a realistic host tissue vascular structure and vessel regression. Similar approach implemented in this work is expected to be repeated for tumor growth in a well vascularized tissue that supports acidosis-free tumor growth over a longer time and with simulations that are conducted over larger domain sizes and longer time intervals. A significant underlying assumption of the current model is the metabolic homogeneity of cancer cells. Evolutionary and ecological dynamics of cancer give rise to specialized niches of neoplastic cells which may exhibit differential metabolic strategies^41,42^. Using the EGT, Lloyd *et al*.^42^ predicted differential cancer cell phenotypic properties at the tumor core and periphery. The authors also reported higher levels of glucose transporter GLUT1 expression at the tumor periphery with respect to tumor core in human stage 2 invasive breast cancers. On the other hand, the incorporation of metabolic heterogeneity in previous spatial computational models showed that glycolytic, acid producing phenotypes constitute a major fraction of tumors^27,43^. The relative abundance of highly glycolytic tumors with respect to metabolically near normal cells was also observed in TRAMP mouse models of prostate cancer^43^. Therefore, attributing a uniform glucose uptake rate to a tumor may be a suitable simplifying assumption while this assumption needs further investigation.

Previous models have incorporated normal tissue cells into their models^17,20,27^ where normal cells act as a spatial constraint and retard tumor progression but are eventually repelled due to acid accumulation. The net effect of the inclusion of normal cells is, therefore, a delay in tumor outgrowth which only requires simulation of tumor growth over longer times with minimal impact on final results. A more meaningful complexity expected to be incorporated into the present model is related to the metabolic interaction between the tumor and its stroma^44–46^ via incorporating stromal cells into the tumor microenvironment. The model could also be extended to three-dimensional (3D) space to provide a more realistic illustration of tumor morphology and growth over time. Nevertheless, given the high computational cost of 3D agent-based models, the current 2D model is cost effective in recapitulating key events occurring as a result of the presence of Warburg effect within the tumor microenvironment. Another future extension to the developed computational model is to incorporate immune cells into the model and determine how the Warburg effect contributes to immune suppression via acidification and nutrient depletion. Additionally, the current model could be implemented to optimize the efficacy and investigate dose dependency of the so-called pH buffering therapies^23,24^.

In summary, the computational model developed in this work elucidates the function of Warburg effect in the context of tumor microenvironment and determines what possible advantages it can confer to cancer cells. We present an *in silico* model of tumor microenvironment capable of predicting various aspects of tumor growth including tumor morphology, cellularity, vascularity, and size. The model also depicts the role played by the glucose metabolism in determining microenvironmental acidity, competition over glucose resources, and possible resistance to anti-angiogenic therapies.

## Methods

### Model overview

**Figure 6:**
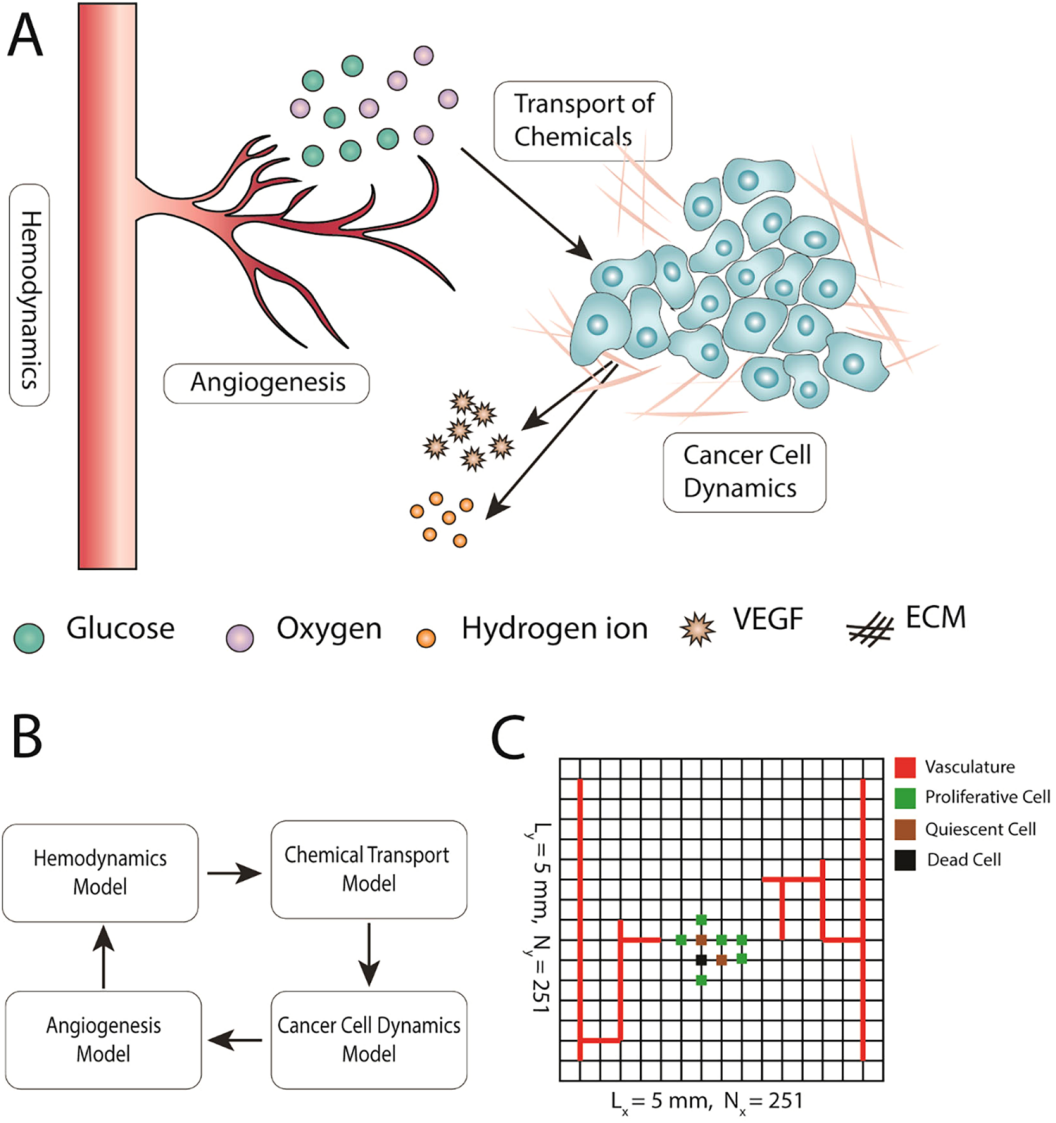
Overview of the multi-scale model of tumor microenvironment to investigate how the glucose uptake rate of cancer cells affects tumor growth. **(A)** Among manifold constituents of the tumor microenvironment, the proposed model opts for incorporation of cancer cells, extracellular matrix (ECM), vasculature, and certain diffusible species. **(B)** To consider the dynamics of each model element, the model consists of four primary compartments accounting for tumor cell dynamics, angiogenesis, hemodynamics, and transport of chemicals in the tumor microenvironment. Every compartment may also involve sub-compartments which are not depicted here for brevity. **(C)** Computational modeling is implemented on a 5 mm by 5 mm square domain containing two parallel parent vessel segments on each side. To trace cancer cell dynamics on a single cell level, the domain is discretized into 251 × 251 nodes. Each node may be either empty or occupied by cancer cells. The state of cells (proliferative, quiescent or dead) are color coded. Because of the single cell resolution of the angiogenesis model, vasculature points are also defined on the same grid.

To keep the complexity of the model at a tractable level, we incorporated only certain elements of the tumor microenvironment in the developed model, including cancer cells, vasculature, the extracellular matrix (ECM), and certain diffusible chemicals (Fig. 6A). The proposed tumor model consists of four primary compartments accounting for the tumor cell dynamics (i.e. cell cycle and metabolism), sprouting angiogenesis, hemodynamics, and transport of diffusible molecules to incorporate the aforementioned tumor microenvironment elements and their dynamics (Fig. 6B). The simulation domain is a 5 mm × 5 mm square (Fig. 6C). Since the cellular automaton (CA) model treats the cell population at a single cell resolution, it requires that each grid node contains a single cancer cell. Consulting the literature^47–49^, a grid size of Δx = Δy = 20 µm was set which corresponds to the approximate size of a tumor cell. Given the single cell resolution of the angiogenesis model, the same grid of the tumor domain is used to solve the angiogenesis model. The governing equations of the tumor compartments are briefly presented below while a detailed description of equations along with the model parameters are presented in Supplementary Information (SI).

### Transport of chemicals

Diffusible chemicals incorporated into the model include oxygen and glucose as nutrients, vascular endothelial growth factor (VEGF) as a tumor angiogenic factor, and hydrogen ions as metabolic waste (Fig. 6A). Each tumor cell is assumed to consume nutrients and excrete metabolic waste. Tumor cells also secrete VEGF under hypoxic conditions. *C*_*m*_(***r***, *t*) denotes the concentration of diffusible molecule *m* at location ***r*** and time *t* in the interstitium. Diffusive transport of *m* in the interstitial space is modeled in Equation (1)

1 $$\frac{\partial {C}_{m}}{\partial t}={D}_{m}{\nabla }^{2}{C}_{m}+{R}_{m}$$

where *R*_*m*_ denotes the sum of source and sink terms originating from cellular and vascular contributions to the field of diffusible molecule *m*. Blood was taken to be a biphasic mixture of erythrocytes and plasma. The total concentration of oxygen in the blood *C*_*b*,*O*2_ is thus determined by adding the plasma oxygen concentration *α*_*b*_*P*_*b*_ with the hemoglobin-bound oxygen concentration *C*_*Hb*_*SH*_*D*_ as ${C}_{b,O2}={\alpha }_{b}{P}_{b}+{C}_{Hb}S{H}_{D}$^50^ where *α*_*b*_ is blood oxygen solubility, *P*_*b*_ is blood oxygen tension, *C*_*Hb*_ is oxygen binding capacity of hemoglobin, *S* is hemoglobin oxygen saturation, and *H*_*D*_ is capillary hematocrit. Using the well-known Hill equation $S({P}_{b})={P}_{b}^{n}/({P}_{b}^{n}+{P}_{50}^{n})$ as a correlation between *S* and *P*_*b*_ (where *P*_50_ is the half saturation oxygen partial pressure and *n* is the Hill exponent), one can substitute for *P*_*b*_ in terms of *S* in the total oxygen concentration relation and arrive at a relation for *C*_*b*,*O*2_ written in terms of *S*. Thus, the capillary oxygen transport can be written in terms of hemoglobin oxygen saturation *S* as described in Equation (2).

2 $$\frac{\partial S}{\partial t}+{{\boldsymbol{v}}}_{{\boldsymbol{b}}}.\nabla S=N(S)$$

where *N*(*S*) is a nonlinear function of *S* with its non-linearity stemming from the biphasic nature of blood. The reader is referred to Section S.3.1 of the SI for a full derivation of Equation (2) and the exact form of the non-linear function *N*(*S*).

### Angiogenesis and hemodynamics

As shown in Fig. 6A, two adjacent vessels supply the tissue with metabolites and take away metabolic waste. Endothelial cells (ECs) lining the vessel walls sprout in response to the VEGF signals released by hypoxic tumor cells and migrate in the direction of VEGF gradients while interacting with the ECM constituents^51,52^. Among the ECM constituents (e.g. collagens, elastins, fibronectins and laminins), fibronectin is a key player in terms of modulating cell attachment, function, and migration^53^ and is incorporated in the current model to recapitulate essential EC-ECM interactions. Sprouting of tip ECs from preexisting parent vessels at position ***r*** and time *t* is assumed to occur with a VEGF dependent probability ${P}_{sprout}={P}_{sprout}({C}_{v}({\boldsymbol{r}},t))$. The positions of EC tips are updated with a random walk formulation, biased by VEGF and fibronectin concentrations, obtained from discretizing Equation (3) on a 2D Cartesian grid^51^.

3 $$\frac{\partial e}{\partial t}={D}_{e}{\nabla }^{2}e-\nabla .(\frac{\chi }{1+\sigma {C}_{v}}e\nabla {C}_{v})-\rho \nabla .(e\nabla f)$$

where *e*(***r***, *t*) and *f*(***r***, *t*) denote non-dimensional EC density and fibronectin concentration, respectively. *D*_*e*_, *χ*, and *ρ* are non-dimensional EC diffusion, chemotaxis, and haptotaxis coefficients, respectively. The well-known Poiseuille relation is applied to determine blood flow *Q* and hematocrit *H*_*D*_ while blood viscosity is defined as a function of capillary diameter and hematocrit ($\mu =\mu (d,{H}_{D})$). Moreover, mass conservation equations at microvascular network nodes are imposed upon the total blood flow *Q* and erythrocyte flow $\overline{Q}=Q{H}_{D}$, and are complimented by empirical equations of hematocrit distribution at the nodes of the microvascular network. A non-linear problem is solved to determine *Q* and *H*_*D*_ over the capillary network (see Fig. S1).

### Cancer cell dynamics

**Figure 7:**
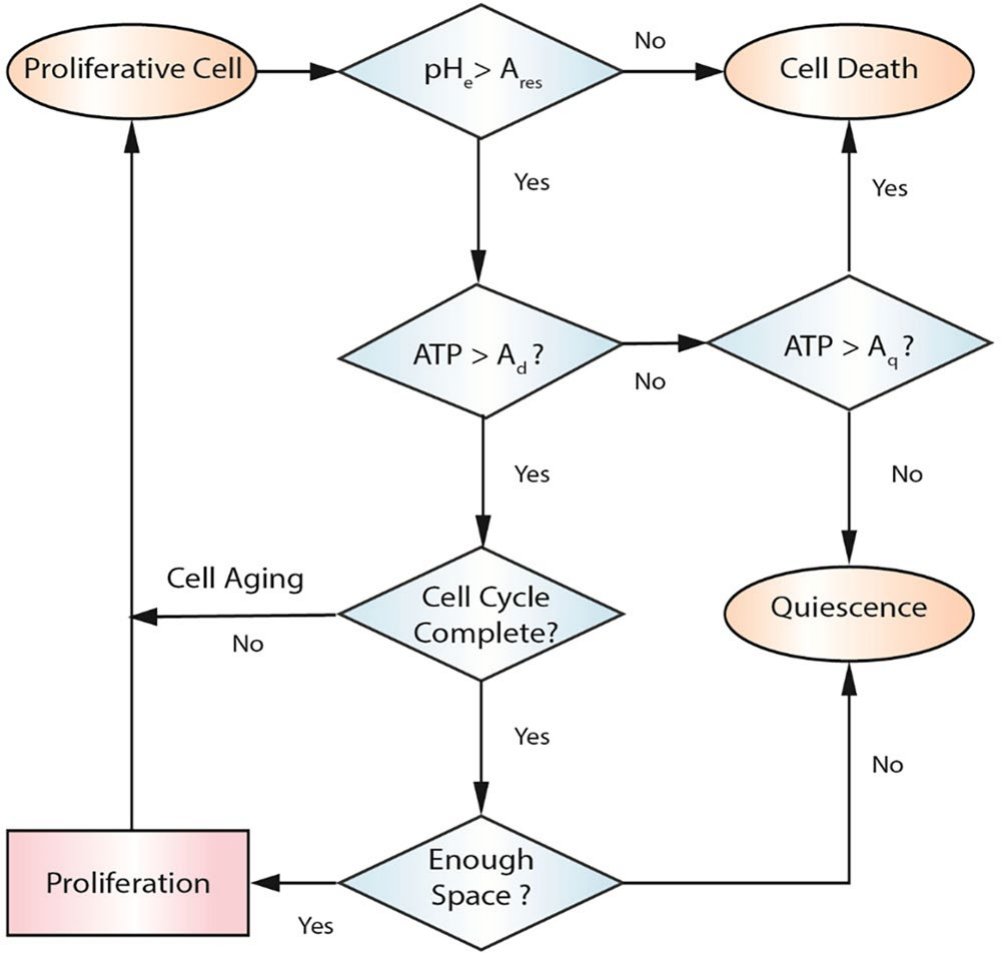
Cellular automaton rules considered to update cell state. Microenvironmental pH, amount of ATP produced by cancer cells, and availability of space for dell division are determinants of cell state. Defining the normalized ATP production rate as $AT{P}^{\ast }={\omega }_{A}/{A}_{0}$, a proliferative cell dies due to necrosis when it does not meet the ATP threshold for death *A*_*d*_. If cell’s ATP yield lies between ATP thresholds for death *A*_*d*_ and quiescence *A*_*q*_, it would undergo quiescence. A quiescent cell returns to proliferative state when its ATP production level is restored to above *A*_*q*_. Denoting the acid resistance of cells as *A*_*res*_, a cell dies due to apoptosis once the extracellular pH falls below the cell acid resistance (*pH*_*e*_ < *A*_*res*_). The acid resistance of cancer cells is set to pH = 6^17^. If a proliferative cell passes the pH and ATP requirements, it would age until the cell cycle is completed. Upon completion of the cell cycle, the cell duplicates which is an indication for the existence of adjacent empty grid elements; otherwise, the cell becomes quiescent. Upon duplication, one of daughter cells remains on the current grid node while the other one occupies randomly an empty adjacent grid point. The status of cancer cells at each time step is updated randomly to avoid spatial bias in tumor growth.

We adopted a recently developed minimal model of cellular metabolism to incorporate core metabolic activities, i.e. glycolysis, citric acid cycle and oxidative phosphorylation^27,43^. Assuming that oxygen consumption obeys the Michaelis–Menten kinetics, the rate of oxygen uptake is computed as ${\omega }_{O2}=-\,{V}_{O2}\{{C}_{O2}/({C}_{O2}+{K}_{O2})\}$ where ${\omega }_{O2}$ is oxygen uptake rate, *C*_*O*2_ is oxygen concentration, *K*_*O*2_ is the half maximum oxygen concentration, and *V*_*O*2_ is the maximum oxygen consumption. Glucose consumption rate ${\omega }_{G}$ is also computed in Equation (4)^43^.

4 $${\omega }_{G}=-(\frac{1}{2}{p}_{G}{A}_{0}+\frac{27}{10}{\omega }_{O2})\frac{{C}_{G}}{{C}_{G}+{K}_{G}}$$

where ${A}_{0}=29\cdot {V}_{O2}/5$ is the baseline production rate of ATP, and *C*_*G*_ and *K*_*G*_ are defined as glucose concentration, and half maximum concentration, respectively. The Warburg effect is included in the model with the factor *p*_*G*_, where *p*_*G*_ = 1 corresponds to normal glucose consumption of cancer cells and *p*_*G*_ > 1 denotes the upregulated glucose uptake rate. Moreover, the term $({p}_{G}\cdot {A}_{0}/2+27\cdot {\omega }_{O2}/10)$ includes the Pasteur effect since more glucose is consumed at lower oxygen uptake rates. With *ω*_*O*2_ and *ω*_*G*_ computed, the ATP production rate *ω*_*A*_ and proton disposal rate *ω*_*H*_ are calculated as ${\omega }_{A}=-\,(2{\omega }_{G}+27\cdot {\omega }_{O2}/5)$ and ${\omega }_{H}=29\cdot ({p}_{G}{V}_{O2}+{\omega }_{O2})\cdot {k}_{H}/5$, respectively, where ${k}_{H} < 1$ accounts for proton buffering in the tumor microenvironment. A set of predefined rules (cellular automaton rules) are used to update cell status in each time step^27,54^. The CA model is essentially a set of decision making processes applied to each cell within the domain. Available space for cell division, microenvironmental pH, and amount of ATP produced by cancer cells are determinants of the cells state (Fig. 7).

## Electronic supplementary material

Supplementary Info

## References

[1] Gatenby RA, Gawlinski ET (2003). The glycolytic phenotype in carcinogenesis and tumor invasion. Cancer Research.

[2] Liberti MV, Locasale JW (2016). The Warburg effect: how does it benefit cancer cells?. Trends in Biochemical Sciences.

[3] Vander Heiden MG, Cantley LC, Thompson CB (2009). Understanding the Warburg effect: the metabolic requirements of cell proliferation. Science.

[4] Stark H, Fichtner M, König R, Lorkowski S, Schuster S (2015). Causes of upregulation of glycolysis in lymphocytes upon stimulation. A comparison with other cell types. Biochimie.

[5] Gatenby RA, Gillies RJ (2004). Why do cancers have high aerobic glycolysis?. Nature Reviews Cancer.

[6] Estrella V (2013). Acidity generated by the tumor microenvironment drives local invasion. Cancer Research.

[7] Kareva I, Hahnfeldt P (2013). The emerging “hallmarks” of metabolic reprogramming and immune evasion: distinct or linked?. Cancer Research.

[8] Chang C-H (2015). Metabolic competition in the tumor microenvironment is a driver of cancer progression. Cell.

[9] Lyssiotis CA, Kimmelman AC (2017). Metabolic interactions in the tumor microenvironment. Trends in Cell Biology.

[10] Schuster S, Boley D, Möller P, Stark H, Kaleta C (2015). Mathematical models for explaining the Warburg effect: a review focussed on ATP and biomass production. Biochemical Society Transactions.

[11] Vazquez A, Liu J, Zhou Y, Oltvai ZN (2010). Catabolic efficiency of aerobic glycolysis: the Warburg effect revisited. BMC Systems Biology.

[12] Basanta D, Simon M, Hatzikirou H, Deutsch A (2008). Evolutionary game theory elucidates the role of glycolysis in glioma progression and invasion. Cell Proliferation.

[13] Kareva I (2011). Prisoner’s dilemma in cancer metabolism. PloS One.

[14] Archetti M (2014). Evolutionary dynamics of the Warburg effect: glycolysis as a collective action problem among cancer cells. Journal of Theoretical Biology.

[15] Hummert S (2014). Evolutionary game theory: cells as players. Molecular BioSystems.

[16] Gatenby RA, Gawlinski ET (1996). A reaction-diffusion model of cancer invasion. Cancer Research.

[17] Patel AA, Gawlinski ET, Lemieux SK, Gatenby RA (2001). A cellular automaton model of early tumor growth and invasion: the effects of native tissue vascularity and increased anaerobic tumor metabolism. Journal of Theoretical Biology.

[18] Smallbone K, Gavaghan DJ, Gatenby RA, Maini PK (2005). The role of acidity in solid tumour growth and invasion. Journal of Theoretical Biology.

[19] Gatenby RA, Gawlinski ET, Gmitro AF, Kaylor B, Gillies RJ (2006). Acid-mediated tumor invasion: a multidisciplinary study. Cancer Research.

[20] Smallbone K, Gatenby RA, Gillies RJ, Maini PK, Gavaghan DJ (2007). Metabolic changes during carcinogenesis: potential impact on invasiveness. Journal of Theoretical biology.

[21] Gatenby R (2007). Cellular adaptations to hypoxia and acidosis during somatic evolution of breast cancer. British Journal of Cancer.

[22] Kareva I, Berezovskaya F (2015). Cancer immunoediting: a process driven by metabolic competition as a predator–prey–shared resource type model. Journal of Theoretical Biology.

[23] Martin N (2012). Predicting the safety and efficacy of buffer therapy to raise tumour pHe: an integrative modelling study. British Journal of Cancer.

[24] Silva AS, Yunes JA, Gillies RJ, Gatenby RA (2009). The potential role of systemic buffers in reducing intratumoral extracellular pH and acid-mediated invasion. Cancer Research.

[25] Archetti M (2015). Heterogeneity and proliferation of invasive cancer subclones in game theory models of the Warburg effect. Cell Proliferation.

[26] Kaznatcheev A, Vander Velde R, Scott JG, Basanta D (2017). Cancer treatment scheduling and dynamic heterogeneity in social dilemmas of tumour acidity and vasculature. British Journal of Cancer.

[27] Robertson-Tessi M, Gillies RJ, Gatenby RA, Anderson AR (2015). Impact of metabolic heterogeneity on tumor growth, invasion, and treatment outcomes. Cancer Research.

[28] Kallinowski F (1988). Glucose uptake, lactate release, ketone body turnover, metabolic micromilieu, and pH distributions in human breast cancer xenografts in nude rats. Cancer Research.

[29] Chen Y, Wang H, Zhang J, Chen K, Li Y (2015). Simulation of avascular tumor growth by agent-based game model involving phenotype-phenotype interactions. Scientific Reports.

[30] Calcinotto A (2012). Modulation of microenvironment acidity reverses anergy in human and murine tumor-infiltrating T lymphocytes. Cancer Research.

[31] Gerweck LE, Vijayappa S, Kozin S (2006). Tumor pH controls the *in vivo* efficacy of weak acid and base chemotherapeutics. Molecular Cancer Therapeutics.

[32] Pilon-Thomas S (2016). Neutralization of tumor acidity improves antitumor responses to immunotherapy. Cancer Research.

[33] Kunkel M (2003). Overexpression of Glut‐1 and increased glucose metabolism in tumors are associated with a poor prognosis in patients with oral squamous cell carcinoma. Cancer.

[34] Jang SM (2012). The glycolytic phenotype is correlated with aggressiveness and poor prognosis in invasive ductal carcinomas. Journal of Breast Cancer.

[35] Soleimani S (2018). Translational models of tumor angiogenesis: A nexus of *in silico* and *in vitro* models. Biotechnology Advances.

[36] Byrne HM (2010). Dissecting cancer through mathematics: from the cell to the animal model. Nature Reviews Cancer.

[37] Nardo G (2011). Glycolytic phenotype and AMP kinase modify the pathologic response of tumor xenografts to VEGF neutralization. Cancer Research.

[38] Curtarello M (2015). VEGF-targeted therapy stably modulates the glycolytic phenotype of tumor cells. Cancer Research.

[39] Yankeelov TE, Abramson RG, Quarles CC (2014). Quantitative multimodality imaging in cancer research and therapy. Nature Reviews Clinical Oncology.

[40] Yankeelov TE (2013). Clinically relevant modeling of tumor growth and treatment response. Science translational medicine.

[41] Gatenby RA, Gillies RJ (2008). A microenvironmental model of carcinogenesis. Nature Reviews Cancer.

[42] Lloyd MC (2016). Darwinian dynamics of intratumoral heterogeneity: not solely random mutations but also variable environmental selection forces. Cancer Research.

[43] Ibrahim-Hashim A (2017). Defining cancer subpopulations by adaptive strategies rather than molecular properties provides novel insights into intratumoral evolution. Cancer Research.

[44] Martinez-Outschoorn UE (2011). Stromal–epithelial metabolic coupling in cancer: integrating autophagy and metabolism in the tumor microenvironment. The international Journal of Biochemistry & Cell Biology.

[45] DeBerardinis RJ (2012). Good neighbours in the tumour stroma reduce oxidative stress. Nature Cell Biology.

[46] Hulikova A (2016). Stromal uptake and transmission of acid is a pathway for venting cancer cell-generated acid. Proceedings of the National Academy of Sciences.

[47] Lesart A-C, Van Der Sanden B, Hamard L, Estève F, Stéphanou A (2012). On the importance of the submicrovascular network in a computational model of tumour growth. Microvascular Research.

[48] Anderson AR (2005). A hybrid mathematical model of solid tumour invasion: the importance of cell adhesion. Mathematical Medicine and Biology.

[49] Cai Y, Wu J, Li Z, Long Q (2016). Mathematical modelling of a brain tumour initiation and early development: a coupled model of glioblastoma growth, pre-existing vessel co-option, angiogenesis and blood perfusion. PloS One.

[50] Goldman D (2008). Theoretical models of microvascular oxygen transport to tissue. Microcirculation.

[51] Anderson AR (1998). & Chaplain, M. Continuous and discrete mathematical models of tumor-induced angiogenesis. Bulletin of Mathematical Biology.

[52] Anderson, A. R., Chaplain, M. A. & McDougall, S. In *Modeling Tumor Vasculature* 105–133 (Springer 2012).

[53] Frantz C, Stewart KM, Weaver VM (2010). The extracellular matrix at a glance. J Cell Sci.

[54] Gerlee P, Anderson AR (2008). A hybrid cellular automaton model of clonal evolution in cancer: the emergence of the glycolytic phenotype. Journal of Theoretical Biology.

