# Supplementary material for: Mathematical Modeling of the Function of Warburg Effect in Tumor Microenvironment
Source: Sci Rep. 2018 Jun 11;8:8903. doi: 10.1038/s41598-018-27303-6 (PMC5995918; doi:10.1038/s41598-018-27303-6)
Supplement: Supplementary file 1 — Supplementary Info [file 41598_2018_27303_MOESM1_ESM.docx]

**Mathematical Modeling of the Function of Warburg Effect in Tumor Microenvironment**

Milad Shamsi^1,2^, Mohsen Saghafian^2^, Morteza Dejam^3^, and Amir Sanati-Nezhad1,4[[1]](#footnote-1)†

*^1^ Center for BioEngineering Research and Education, University of Calgary, Calgary, Alberta T2N 1N4, Canada*

*^2^ Department of Mechanical Engineering, Isfahan University of Technology, Isfahan 8415683111, Iran*

*^3^ Department of Petroleum Engineering, College of Engineering and Applied Science, University of Wyoming, 1000 E. University Avenue, Laramie, Wyoming 82071–2000, USA*

*^4^ BioMEMS and Bioinspired Microfluidic Laboratory, Department of Mechanical and Manufacturing Engineering, University of Calgary, Calgary, Alberta T2N 2N1, Canada*

**Supplemental Information**

Herein, we present further details of the computational model and the simulation methods. The model parameters are also included here.

**S.1. Angiogenesis model**

Endothelial cells (ECs), lining the walls of parent vessels, are assumed to detach and form angiogenic sprouts upon receiving vascular endothelial growth factor (VEGF) signals from hypoxic cancer cells. Sprouting is assumed to initiate once VEGF concentration $C_{v}$ exceeds a definite threshold $C_{th}$, with the sprouting probability computed as Equation S1.1.^1^

| (S1.1) | $P_{sprout}=\left\{ \begin{aligned} {k_{p}l}_{seg}\Delta t \left( \frac{C_{v}-C_{th}}{C_{v}-C_{th}+C_{th,50}} \right),C_{v}>C_{th} \\ 0 ,C_{v}<C_{th} \end{aligned} \right.$ |
| --- | --- |

where $k_{p}$ is the maximal probability of sprout formation per length per time, $l_{seg}$ is the vessel segment length, and $\Delta t$ is the time step size. The term in parentheses delineates increased likeliness of sprouting at higher VEGF concentrations where $C_{th,50}$ is a parameter presented in **Table. S1.**

The well-known model of Anderson and Chaplain^2,3^ was adopted to describe angiogenic sprout elongation. The model assumes that the EC’s tip migrates through random motility, with chemotaxis in response to VEGF gradients, and with haptotaxis in response to matrix fiber gradients. The non-dimensional equation governing the distribution of ECs is defined as Equation S1.2.^3^

| (S1.2) | $\frac{\partial e}{\partial t}=D_{e}\nabla^{2}e-\nabla.(\frac{\chi}{1+\sigma C_{v}}e\nabla C_{v})-\rho\nabla.(e\nabla f)$ |
| --- | --- |

where $e$, $f$, and $C_{v}$ denote non-dimensional EC density, fibronectin concentration and VEGF concentration, respectively. Here $D_{e}$ , $\chi$, and $\rho$are non-dimensional EC diffusion, chemotaxis coefficient and haptotaxis coefficient, respectively. Discretizing Equation S1.2 with the Euler finite difference approximation leads to a biased random walk formulation for the displacement of each EC’s tip on a 2D Cartesian grid. The discretized Equation S1.2 is solved on the computational grid to update the tip location at each time step. Branching and anastomosis are also introduced into the model as described in somewhere else.^2^ Moreover, the interaction of sprouts and the ECM fibers is phenomenologically described with Equation S1.3.

| $\frac{\partial f}{\partial t}=\delta e-\kappa ef$ | (S1.3) |
| --- | --- |

where $\delta$ and $\kappa$ are positive constants expressing fibronectin production and proteolytic activity of EC tip, respectively. Since the diameter of most tumor microvessels lies within the range $10<d<20\mu m$,^4,5^ the value of capillary diameter is assigned to $d=15\mu m$ for every vessel of the network.

**S.2. Hemodynamics model**

After updating the microvascular structure, hemodynamic calculations are solved to determine the flow rate and hematocrit value in every capillary segment, which are subsequently used to compute intravascular concentrations of oxygen in every vessel segment. The flow rate in each capillary segment is determined by imposing the mass conservation equation at each node of the microvascular network (Equation S2.1).^6^

| (S2.1) | $\sum Q_{c,k}=0$ |
| --- | --- |

where $Q_{c,k}$ is the blood volumetric flow rate between the current node $c$and each of its adjacent nodes $k$. Because of low Reynolds number ($\mathrm{Re}<1$) in capillaries, the Hagen-Poiseuille equation is applied to calculate the intracapillary flow $Q_{c,k}$ (Equation S2.2).^6^

| (S2.2) | $Q_{c,k}=\frac{\pi}{128}\frac{\Delta P.d^{4}}{\mu L}$ |
| --- | --- |

where $\mu$, $L$and $d$ are blood viscosity, capillary segment length and capillary segment diameter, respectively. To account for the biphasic nature of the blood, the conservation of erythrocyte flow is considered at each node (Equation S2.3).^7^

| (S2.3) | $\sum\overline{Q_{c,k}}=0$ |
| --- | --- |

where $\overline{Q}=QH_{D}$ is the erythrocyte flow rate and $H_{D}$ is the capillary discharge hematocrit.

***S.2.1 Blood rheology***

The particulate behavior of erythrocytes is no longer neglected as the vessel diameter becomes comparable to that of erythrocytes.^8^ Therefore, the blood is regarded as a biphasic fluid with non-Newtonian properties (i.e. blood viscosity is no longer assumed constant) in microvessels. Nonetheless, Hagen-Poiseuille law is still valid for the intracapillary flow where the viscosity $\mu$ in Equation S2.2 is replaced with the so-called apparent viscosity$\mu_{app}$. Defining the relative blood viscosity as the ratio of apparent viscosity to plasma viscosity ($\mu_{rel}=\mu_{app}/\mu_{pl}$), the dependence of $\mu_{rel}$ on capillary diameter and hematocrit is described as Equation S2.4.^9^

| $\mu_{rel}=\left(1+\left(\mu_{45}-1\right)\times\frac{\left(1-H_{D}\right)^{C}}{\left(1-0.45\right)^{C}-1}\times\left(\frac{d}{d-1.1}\right)^{2}\right)\times\left(\frac{d}{d-1.1}\right)^{2}$ | (S2.4) |
| --- | --- |

where *d* is vessel diameter ($\mu m$) and$\mu_{pl}=1.2\times10^{-3} \text{Pa}.\text{s}$. Moreover, $\mu_{45}$ is the relative viscosity corresponding to $H_{D}=0.45$ and is defined in Equation S2.5.

| (S2.5) | $\mu_{45}=3.2+6e^{-0.085d}-2.44e^{-0.06d^{0.645}}$ |
| --- | --- |

where *C* is given by Equation S2.6.

| (S2.6) | $C=\left(0.8+e^{-0.075d}\right)\left(-1+\frac{1}{1+10^{-11}d^{12}}\right)+\frac{1}{1+10^{-11}d^{12}}$ |
| --- | --- |

***S.2.2 Hematocrit distribution at capillary bifurcations***

Capillary hematocrit is a vital factor that affects oxygenation capacity of each microvessel and thereby determines oxygen distribution within the tumor microenvironment. Hematocrit distribution within the microvascular network is heterogeneous which attributes to phase separation at diverging capillary bifurcations where a feeding parent vessel branches into two daughter vessels.^8^ The experimental work of Pries and coworkers^10^ quantified the dependence of fractional erythrocyte flow$FQ_{E}$on fractional blood flow$FQ_{B}$at diverging capillary bifurcations. This dependency is expressed with the empirical relation given by Equation S2.7.^11^

| (S2.7) | ${FQ}_{E}=0$ $if {FQ}_{B}\leq X_{0}$ ${logit FQ}_{E}=A+B.logit (\frac{{FQ}_{B}-X_{0}}{1-2X_{0}})$ $if X_{0}\leq{FQ}_{B}\leq{1-X}_{0}$ ${FQ}_{E}=1$ $if {1-X}_{0}\leq{FQ}_{B}$ |
| --- | --- |

where $logit x=\ln(x/(1-x))$ and parameters *A*, *B*, and $X_{0}$ are given by Equations S2.8 to S2.10.

| (S2.8) | $A=-\frac{13.29(1-H_{D})}{d_{p}}.\frac{(d_{1}/d_{2})^{2}-1}{(d_{1}/d_{2})^{2}+1}$ |
| --- | --- |
| (S2.9) | $B=1+\frac{6.98(1-H_{D})}{d_{p}}$ |
| (S2.10) | $X_{0}=\frac{0.964(1-H_{D})}{d_{p}}$ |

where $d_{1}$ and $d_{2}$ are diameters of daughter vessels, and $d_{p}$ denotes diameter of the parent vessel. All capillary segments are assumed to be of equal size ($d=15\mu m$), and thus the parameter *A* is assumed to be zero. The theoretical extension to the distribution of red blood cells at network trifurcations is also available in the literature.^6^ Equations S2.1 to S2.10 form a coupled system of equations and an iterative method as prescribed somewhere else^12^ is adopted to solve for hematocrit and volumetric flow rates within the network. The algorithm used for calculating the hematocrit and flow rate is shown in **Fig. S1**.

| 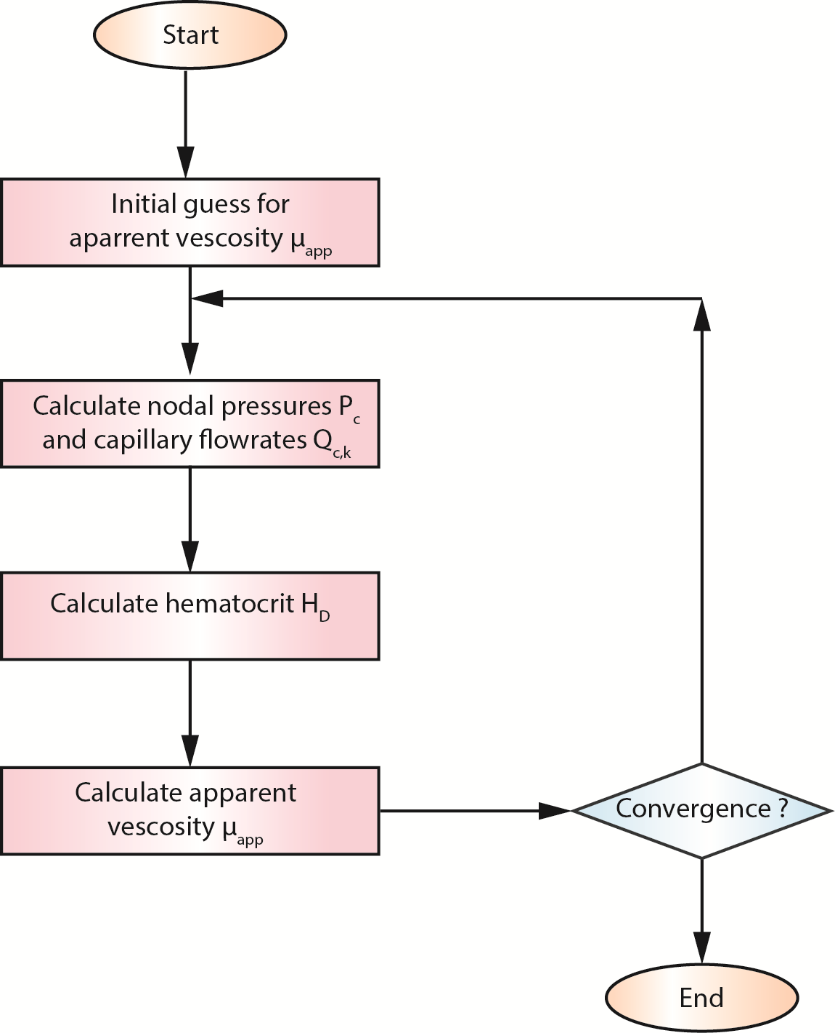 |
| --- |
| **Figure S1.** The algorithm used for calculating the network hematocrit and flow rate. An iterative scheme is adopted to solve the nonlinear problem of the blood flow in capillaries. Following the initial guess of the apparent viscosity, Equations S2.1 and S2.2 are used to calculate the flow rate and nodal pressures of the network, followed by computing the capillary hematocrit using Equation S2.3 as well as Equations S2.7 to S2.10. The blood viscosity for each capillary segment is then recalculated and compared to the initial viscosity from the previous iteration for convergence analysis. This process is iterated until convergence is achieved. |

**S.3. Chemical transport model**

Introduction of certain diffusible chemicals, namely oxygen, glucose, hydrogen ions and VEGF mediate the tumor-stroma coupling. It is assumed that nutrients are delivered to the interstitial space via mass transfer from the intracapillary space. Cancer cells consume nutrients while they produce metabolic waste carried away by the capillary blood flow. It is also assumed that the oxygen concentration varies along the vessel due to mass transfer across the vessel lumen. Intravascular concentrations of glucose and hydrogen ions are, however, preserved throughout the vasculature. Moreover, under hypoxic conditions, cancer cells and ECs that line the wall of vasculature act as sources and sinks for the tumor angiogenic factors, respectively. The governing equations for the chemical transport model are described below.

***S.3.1 Oxygen transport***

$C_{O2}(r,t)$ denotes the concentration of oxygen at location ***r*** and time *t* in the interstitium. Diffusive transport of oxygen in the interstitial space is modeled by Equation S3.1.

| (S3.1) | $\frac{\partial C_{O2}}{\partial t}=D_{O2}\nabla^{2}C_{O2}+a_{O2}+b_{O2}$ |
| --- | --- |

where $a_{O2}$ and $b_{O2}$ are contributions of tumor cells and vasculature to the field of oxygen, respectively and $D_{O2}$ is diffusivity of oxygen within the tissue. The contribution of tumor cells in Equation S3.1 is computed as Equation S3.2.

| (S3.2) |  |
| --- | --- |

where $\omega_{O2}$ is the rate of oxygen uptake by cancer cells. Moreover, denotes the density of tumor cells and $\gamma_{P}$ is a Boolean indicator function describing the presence ($\gamma_{P}=1$) or absence ($\gamma_{P}=0$) of a cancer cell on a grid point *P*. The contribution of vasculature is also determined as Equation S3.3.

| (S3.3) | $b_{O2}=\sum\frac{4q_{O2}}{d}(C_{b,O2}-C_{w,O2})\xi_{P,k}$ |
| --- | --- |

where $q_{O2}$ is the vessel permeability to oxygen (**Table S2**)*.* Also, $C_{b,O2}$ and $C_{w,O2}$ are intravascular and wall concentrations of oxygen. The summation is taken over point *P* and its adjacent nodes (*k=N, S, W, E*). Here $\xi_{P,k}$ is a Boolean indicator denoting the connectivity of *P* with its neighboring nodes. The value of $\xi_{P,k}$ is equal to unity when there exists a capillary segment connecting *P* with *k* and is equal to zero otherwise.

To account for the variation of oxygen along the vessel axis, $C_{b,O2}$ denotes the intravascular concentration of oxygen while Equation S3.4 represents advective transport of oxygen along the vessel.^13^

| (S3.4) | $\frac{\partial C_{b,O2}}{\partial t}+v_{b}.\nabla C_{b,O2}=-\frac{4q_{m}}{d}(C_{b,O2}-C_{w,O2})$ |
| --- | --- |

where $v_{b}$ is blood velocity. Blood was taken to be a biphasic mixture of plasma and erythrocytes. Blood total oxygen is then determined via adding free and hemoglobin bound fractions as Equation S3.5.^14^

| (S3.5) | $C_{b,O2}=\alpha_{b}P_{b}+C_{Hb}SH_{D}$ |
| --- | --- |

where *S* is hemoglobin oxygen saturation defined as the fraction of available oxygen binding sites occupied by oxygen,^15^ $C_{Hb}$ is oxygen binding capacity of hemoglobin, $P_{b}=C_{O2}/\alpha_{b}$ is blood oxygen tension and $\alpha_{b}$ is blood oxygen solubility defined in Equation S3.6.^16^

| (S3.6) | $\alpha_{b}=H_{D}\alpha_{RBC}+(1-H_{D})\alpha_{pl}$ |
| --- | --- |

where $\alpha_{pl}$ and $\alpha_{RBC}$ are oxygen solubility in plasma and red blood cell, respectively. Hemoglobin oxygen saturation *S* and blood oxygen tension $P_{b}$ are correlated as Equation S3.7.^16^

| (S3.7) | $S(P_{b})=\frac{P_{b}^{n}}{P_{b}^{n}+P_{50}^{n}}$ |
| --- | --- |

where $P_{50}$ is half saturation $P_{O2}$ and *n* is Hill exponent (**Table S.2**). Using Equation S3.7, the blood oxygen tension is written as $P_{b}=P_{50}F(S)$ where $F(S)=S^{1/n}/(1-s)^{1/n}$. Substituting for $P_{b}$ in Equation S3.5 yields Equation S3.8.

| $C_{b,O2}=\alpha_{b}P_{50}F(S)+C_{Hb}SH_{D}$ | (S3.8) |
| --- | --- |

Next, we substitute for $C_{b,O2}$from Equation S3.8 into Equation S3.4. To write Equation S3.4 in terms of the hemoglobin oxygen saturation *S,* the first and second terms on the left hand of Equation S3.4 are then written as Equations S3.9 and S3.10, respectively.

| $\frac{\partial C_{b,O2}}{\partial t}=(\alpha_{b}P_{50}\frac{dF(S)}{dS}+C_{Hb}H_{D})\frac{\partial S}{\partial t}=A(S)\frac{\partial S}{\partial t}$ | (S3.9) |
| --- | --- |
| $v_{b}.\nabla C_{b,O2}=v_{b}.(\alpha_{b}P_{50}\frac{dF(S)}{dS}+C_{Hb}H_{D})\nabla S=v_{b}.A(S)\nabla S$ | (S3.10) |

where $\nabla\equiv(\partial/\partial l)e_{l}$, and $l$ is the curvilinear coordinate along the axis of a given capillary. Moreover, $e_{l}$denotes the unit vector of the curvilinear coordinate $l$. Similarly, substituting for $C_{b,O2}$in the right-hand side of Equation S3.4 yields Equation S3.11

| $-\frac{4q_{m}}{d}(C_{b,O2}-C_{w,O2})=-\frac{4q_{m}}{d}(\alpha_{b}P_{50}F(S)+C_{Hb}SH_{D}-C_{w,O2})=B(S)$ | (S3.11) |
| --- | --- |

Finally, integrating Equations S3.9-S3.11 with Equation S3.4 yields a non-linear and time-dependent equation expressing the change of hemoglobin oxygen saturation *S* along each capillary segment.

| (S3.12) | $\frac{\partial S}{\partial t}+v_{b}.\nabla S=N(S)$ |
| --- | --- |

where $N(S)=B(S)/A(S)$ is a non-linear function of *S* with its non-linearity stemming from the biphasic nature of blood incorporated into the developed model. Equations S3.1-S3.7 along with Equation S3.12 form a non-linear, coupled system of equations solved for determining the intravascular and interstitial oxygen concentrations.

***S.3.2 Glucose and acid transport***

The parameters $C_{G}(r,t)$ and $C_{H}(r,t)$denote the concentrations of glucose and hydrogen ion within the tissue, respectively. The equations governing the interstitial transport of glucose and acid are defined as Equations S3.13 and S3.14, respectively.

| (S3.13) | $\frac{\partial C_{G}}{\partial t}=D_{G}\nabla^{2}C_{G}+a_{G}+b_{G}$ |
| --- | --- |
| (S3.14) | $\frac{\partial C_{H}}{\partial t}=D_{H}\nabla^{2}C_{H}+a_{H}+b_{H}$ |

where $D_{G}$ and $D_{H}$ are tissue diffusion coefficients of glucose and hydrogen ions, respectively. Moreover, the contributing terms of tumor cells and vasculature are computed via Equations S3.15 to S3.18.

| (S3.15) |  |
| --- | --- |
| (S3.16) |  |
| (S3.17) | $b_{G}=\sum\frac{4q_{G}}{d}\left(C_{b,G}-C_{w,G}\right). \xi_{P,k}$ |
| (S3.18) | $b_{H}=\sum\frac{4q_{H}}{d}\left(C_{b,H}-C_{w,H}\right). \xi_{P,k}$ |

where $q_{G}$ and $q_{H}$ are vessel wall permeability to glucose and hydrogen ions, respectively. Moreover, glucose uptake rate $\omega_{G}$ and proton production rate $\omega_{H}$ are determined by equations given in the main text. We set constant values for intravascular concentrations of glucose ($C_{b,g}=5\times10^{-3}M$) and acid ($C_{b,H}=3.98\times10^{-8}M$),^17^ and solve Equations S3.13 to S3.18 to determine glucose and pH fields.

***S.3.3 Growth factor transport***

Tumor cells secret VEGF at the rate of $\omega_{v}$ when oxygen tension falls below the hypoxic limit (set to 10 mmHg). VEGF diffuses into the interstitium with the diffusivity$D_{v}$, decays with a natural decay rate of $\lambda_{v}$ and is taken up by ECs with a rate of $\varepsilon_{v}$. Thus, the interstitial VEGF transport equation is defined as Equation S3.15.

| (S3.19) |  |
| --- | --- |

where $\eta_{P}$ and $\theta_{P}$ are Boolean indicators of the existence of hypoxic tumor cells and ECs on a grid point *P*, respectively. Also the density of ECs is set to $\psi=10^{9}cells.cm^{-3}$.

**S.4 Boundary and Initial Conditions**

Three tumor cells with random ages are initially placed at the central region of the domain. The ECM is assumed to be intact at time zero with a non-dimensional fiber density equal to unity. Initial pH value of the tumor microenvironment is set to 7.4. Also the VEGF concentration is set to zero at time zero. An initial oxygen tension of *30 mmHg* is set in the tumor microenvironment. Moreover, the initial concentration of glucose within the tissue is set to the serum normal value of $5\times10^{-3}M$.^17^ The inlet oxygen tension ($P_{O2}$) and hematocrit to the parent vessels are set to 30 mmHg and 0.45, respectively. Also the nodal pressure difference of blood in preexisting vessels is set to *ΔP= 100 mmHg* so that the resulting volumetric flow rate $Q_{b}=45nl/\min$ falls within the physiological limits. No flux boundary conditions are adopted for the interstitial transport equations.^7,18,19^

**S.5 Simulation method**

We set up a *251$\times$ 251* tissue grid seeded with tumor cells and parent vessel segments to solve each model element sequentially for a certain simulation period. The code used to carry out simulations was developed in FORTRAN. The algorithm used for each time step of the model is presented in **Fig. S2**. The algorithm starts with updating the cell state (by solving the governing equations of cellular automation and cellular metabolism) with a time step equal to $\Delta t_{cell}=0.25hr$, close to the values used in literature.^18^ This time step was effective to adequately follow the cancer cell dynamics. When the cell status is updated, the acid and VEGF production rates are calculated, the pH and growth factor fields are updated, and the angiogenesis model is solved.

Assuming an average sprout extension rate of *16 µm/hr,*^20^ the time step of the angiogenesis model is set to $\Delta t_{a}=1.25hr$. After updating the vasculature, the hemodynamics calculations are carried out to determine hematocrit and flow rates in every capillary segment over the network and solve the model of oxygen transport. The final step of the algorithm is to solve the glucose field (**Fig. S2**). All transport equations are solved using finite difference method with an explicit discretization scheme.

**S.6 Model parameters**

Parameters of each model compartment along with their values are listed in **Tables S1**-**S3**. The significance of each model parameter and the corresponding references are also included.

| 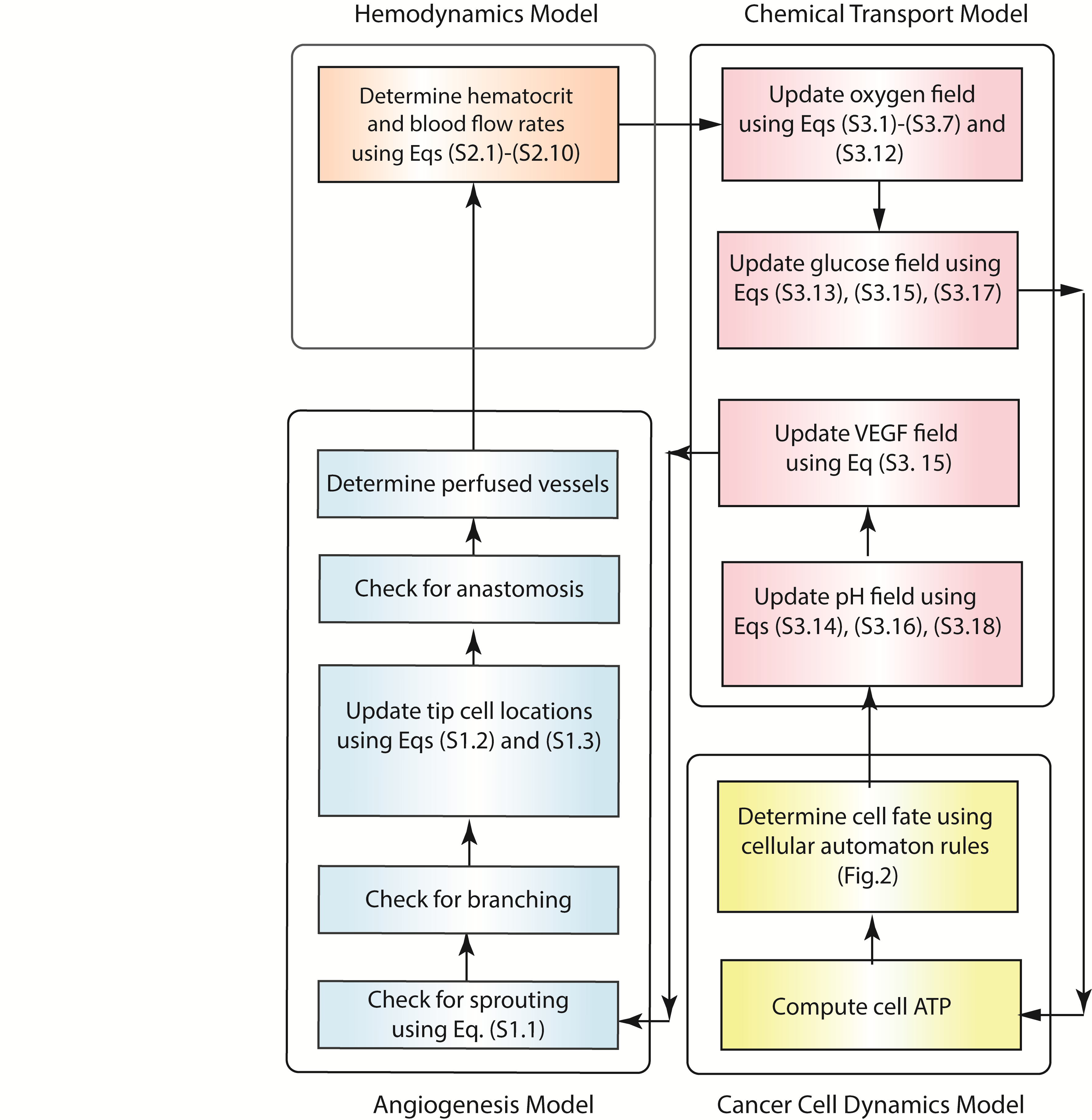 |
| --- |
| **Figure S2.** The algorithm used to update tumor microenvironment status in every timeframe of the simulated model. |

| **Table S1.** Parameters selected for the angiogenesis model. | | | | |
| --- | --- | --- | --- | --- |
| **Parameter** | **Value** | **Unit** | **Significance** | **Ref.** |
| $D_{e}$ | $35\times10^{-5}$ | - | Non-dimensional EC diffusion coefficient | 2 |
| $\chi$ | $0.38$ | - | Non-dimensional EC chemotaxis coefficient | 3 |
| $\rho$ | $0.34$ | - | Non-dimensional EC haptotaxis coefficient | 2 |
| $\kappa$ | $0.1$ | - | Non-dimensional parameter in ECM evolution Eq. (S1.3) | 3 |
| $\delta$ | $0.05$ |  | Non-dimensional parameter in ECM evolution Eq. (S1.3) | 3 |
| $k_{p}$ | $2\times10^{-3}$ | $\mu m^{-1}.day^{-1}$ | Baseline max. sprouting probability | 1 |
| $C_{th}$ | $0.8\times10^{-9}$ | $mol.L^{-1}$ | VEGF threshold for sprouting | 1 |
| $C_{th,50}$ | $0.5\times10^{-9}$ | $mol.L^{-1}$ | Constant in sprouting probability function | 1 |

ECM: Extracellular matrix

VEGF: Vascular endothelial growth factor

| **Table S2.** Parameters of the chemical transport model | | | | |
| --- | --- | --- | --- | --- |
| **Parameter** | **Value** | **Unit** | **Significance** | **Ref.** |
| $\alpha$ | $3.89\times10^{-5}$ | $cm^{3}O_{2}.cm^{-3}.mmHg^{-1}$ | Tissue oxygen solubility | 16 |
| $\alpha_{pl}$ | $2.82\times10^{-5}$ | $cm^{3}O_{2}.cm^{-3}.mmHg^{-1}$ | Plasma oxygen solubility | 16 |
| $\alpha_{RBC}$ | $3.38\times10^{-5}$ | $cm^{3}O_{2}.cm^{-3}.mmHg^{-1}$ | Erythrocyte oxygen solubility | 16 |
| $P_{50}$ | $29.3$ | $mmHg$ | Half-saturation $P_{O2}$ | 16 |
| $n$ | $2.2$ | - | Hill exponent | 16 |
| $C_{Hb}$ | $0.52$ | $cm^{3}O_{2}.cm^{-3}$ | Oxygen binding capacity of hemoglobin | 16 |
| $D_{O2}$ | $2.41\times10^{-5}$ | $cm^{2}.s^{-1}$ | Tissue oxygen diffusivity | 16 |
| $q_{O2}$ | $6.0\times10^{-3}$ | $cm.s^{-1}$ | Vessel permeability to oxygen | 13 |
| $D_{G}$ | $9.0\times10^{-5}$ | $cm^{2}.s^{-1}$ | Tissue glucose diffusivity | 17 |
| $q_{G}$ | $3.0\times10^{-5}$ | $cm.s^{-1}$ | Vessel permeability to glucose | 17 |
| $q_{H}$ | $1.2\times10^{-4}$ | $cm.s^{-1}$ | vessel permeability to hydrogen ions | 17 |
| $D_{H}$ | $1.1\times10^{-5}$ | $cm^{2}.s^{-1}$ | Tissue hydrogen ion diffusivity | 17 |
| $D_{v}$ | $2.9\times10^{-7}$ | $cm^{2}.s^{-1}$ | VEGF diffusion coefficient | 19 |
| $\omega_{v}$ | $2.7\times10^{-24}$ | $mol.cell^{-1}.s^{-1}$ | VEGF production rate by hypoxic TCs | 21 |
| $\varepsilon_{v}$ | $10^{-24}$ | $mol.cell^{-1}.s^{-1}$ | VEGF uptake rate by ECs | - |
| $\lambda_{v}$ | $10^{-6}$ | $s^{-1}$ | VEGF natural decay rate | 22 |

| **Table S3.** Parameter values selected for the model of cancer cell dynamics | | | | |
| --- | --- | --- | --- | --- |
| **Parameter** | **Value** | **Unit** | **Significance** | **Ref.** |
| $V_{O2}$ | $2.3\times10^{-16}$ | $mol.cell.s^{-1}$ | Max. O_2_ consumption | 23 |
| $k_{O2}$ | $5.0\times10^{-6}$ | $mol.L^{-1}$ | Half-max O_2_ concentration | 24 |
| $k_{G}$ | $4.0\times10^{-5}$ | $mol.L^{-1}$ | Half-max glucose concentration | 24 |
| $k_{H}$ | $2.5\times10^{-4}$ | - | Proton buffering coefficient | 25 |
| $A_{res}$ | $6.0$ | $pH$ | Cancer cell acid resistance | 26 |
| $A_{d}$ | $0.3$ | - | ATP threshold for death | 25 |
| $A_{q}$ | $0.8$ | - | ATP threshold for quiescence | 25 |
| $t_{m}$ | $16$ | $hr$ | Mean cell cycle time | 27 |

**References**

1 Secomb, T. W., Alberding, J. P., Hsu, R., Dewhirst, M. W. & Pries, A. R. Angiogenesis: an adaptive dynamic biological patterning problem. *PLoS Comput Biol* **9**, e1002983 (2013).

2 Anderson, A. R. & Chaplain, M. Continuous and discrete mathematical models of tumor-induced angiogenesis. *Bulletin of Mathematical Biology* **60**, 857-899 (1998).

3 Anderson, A. R., Chaplain, M. A. & McDougall, S. in *Modeling Tumor Vasculature* 105-133 (Springer, 2012).

4 Tozer, G. M. *et al.* Intravital imaging of tumour vascular networks using multi-photon fluorescence microscopy. *Advanced Drug Delivery Reviews* **57**, 135-152 (2005).

5 Kim, E. *et al.* Multiscale imaging and computational modeling of blood flow in the tumor vasculature. *Annals of Biomedical Engineering* **40**, 2425-2441 (2012).

6 Soltani, M. & Chen, P. Numerical modeling of interstitial fluid flow coupled with blood flow through a remodeled solid tumor microvascular network. *PloS One* **8**, e67025 (2013).

7 Welter, M., Fredrich, T., Rinneberg, H. & Rieger, H. Computational Model for Tumor Oxygenation Applied to Clinical Data on Breast Tumor Hemoglobin Concentrations Suggests Vascular Dilatation and Compression. *PloS One* **11**, e0161267 (2016).

8 Pries, A., Secomb, T. W. & Gaehtgens, P. Biophysical aspects of blood flow in the microvasculature. *Cardiovascular Research* **32**, 654-667 (1996).

9 Pries, A. *et al.* Resistance to blood flow in microvessels in vivo. *Circulation Research* **75**, 904-915 (1994).

10 Pries, A., Ley, K., Claassen, M. & Gaehtgens, P. Red cell distribution at microvascular bifurcations. *Microvascular Research* **38**, 81-101 (1989).

11 Pries, A. R. & Secomb, T. W. Blood flow in microvascular networks. *Comprehensive Physiology* ***7****(4), 826-34* (2011).

12 Pries, A., Secomb, T. W., Gaehtgens, P. & Gross, J. Blood flow in microvascular networks. Experiments and simulation. *Circulation Research* **67**, 826-834 (1990).

13 Skeldon, A. C. *et al.* Modelling and detecting tumour oxygenation levels. *PloS one* **7**, e38597 (2012).

14 Goldman, D. Theoretical models of microvascular oxygen transport to tissue. *Microcirculation* **15**, 795-811 (2008).

15 Popel, A. S. & Hellums, J. D. Theory of oxygen transport to tissue. *Critical reviews in Biomedical Engineering* **17**, 257 (1989).

16 Goldman, D. & Popel, A. S. A computational study of the effect of capillary network anastomoses and tortuosity on oxygen transport. *Journal of Theoretical Biology* **206**, 181-194 (2000).

17 Gatenby, R. A. & Gawlinski, E. T. The glycolytic phenotype in carcinogenesis and tumor invasion. *Cancer Research* **63**, 3847-3854 (2003).

18 Lesart, A.-C., Van Der Sanden, B., Hamard, L., Estève, F. & Stéphanou, A. On the importance of the submicrovascular network in a computational model of tumour growth. *Microvascular esearch* **84**, 188-204 (2012).

19 Cai, Y., Wu, J., Li, Z. & Long, Q. Mathematical modelling of a brain tumour initiation and early development: a coupled model of glioblastoma growth, pre-existing vessel co-option, angiogenesis and blood perfusion. *PloS One* **11**, e0150296 (2016).

20 Yifat, J. & Gannot, I. 3D discrete angiogenesis dynamic model and stochastic simulation for the assessment of blood perfusion coefficient and impact on heat transfer between nanoparticles and malignant tumors. *Microvascular Research* **98**, 197-217 (2015).

21 Leith, J. & Michelson, S. Secretion rates and levels of vascular endothelial growth factor in clone A or HCT‐8 human colon tumour cells as a function of oxygen concentration. *Cell Proliferation* **28**, 415-430 (1995).

22 Vavourakis, V. *et al.* A Validated Multiscale In-Silico Model for Mechano-sensitive Tumour Angiogenesis and Growth. *Plos Computational Biology* **13**, e1005259 (2017).

23 Molter, T. W. *et al.* A new approach for measuring single-cell oxygen consumption rates. *IEEE Transactions on Automation Science and Engineering* **5**, 32-42 (2008).

24 Ibrahim-Hashim, A. *et al.* Defining cancer subpopulations by adaptive strategies rather than molecular properties provides novel insights into intratumoral evolution. *Cancer Research* **77**, 2242-2254 (2017).

25 Robertson-Tessi, M., Gillies, R. J., Gatenby, R. A. & Anderson, A. R. Impact of metabolic heterogeneity on tumor growth, invasion, and treatment outcomes. *Cancer Research* **75**, 1567-1579 (2015).

26 Patel, A. A., Gawlinski, E. T., Lemieux, S. K. & Gatenby, R. A. A cellular automaton model of early tumor growth and invasion: the effects of native tissue vascularity and increased anaerobic tumor metabolism. *Journal of Theoretical Biology* **213**, 315-331 (2001).

27 Anderson, A. R. A hybrid mathematical model of solid tumour invasion: the importance of cell adhesion. *Mathematical Medicine and Biology* **22**, 163-186 (2005).

1. ^†^Author to whom correspondence should be addressed. Amir Sanati Nezhad, Dept. Mechanical and Manufacturing Engineering, BioMEMS and Bioinspired Microfluidic Laboratory, University of Calgary, EEEL 455B 2500 University Drive NW, Calgary, Alberta, Canada, T2N 1N4, Electronic mail: [amir.sanatinezhad@ucalgary.ca](mailto:amir.sanatinezhad@ucalgary.ca). [↑](#footnote-ref-1)
